# The Active Site of a Prototypical “Rigid” Drug Target is Marked by Extensive Conformational Dynamics

**DOI:** 10.1002/anie.202009348

**Published:** 2020-11-16

**Authors:** Himanshu Singh, Chandan K. Das, Suresh K. Vasa, Kristof Grohe, Lars V. Schäfer, Rasmus Linser

**Affiliations:** ^1^ Faculty of Chemistry and Chemical Biology Technical University Dortmund Otto-Hahn-Str. 4a 44227 Dortmund Germany; ^2^ Faculty for Chemistry and Pharmacy Ludwig-Maximilians-University Munich Butenandtstr. 5–13 81377 Munich Germany; ^3^ Theoretical Chemistry Ruhr University Bochum Universitätsstr. 150 44801 Bochum Germany

**Keywords:** carbonic anhydrase II, conformational exchange dynamics, drug discovery, NMR spectroscopy, protein structure

## Abstract

Drug discovery, in particular optimization of candidates using medicinal chemistry, is generally guided by structural biology. However, for optimizing binding kinetics, relevant for efficacy and off‐target effects, information on protein motion is important. Herein, we demonstrate for the prototypical textbook example of an allegedly “rigid protein” that substantial active‐site dynamics have generally remained unrecognized, despite thousands of medicinal‐chemistry studies on this model over decades. Comparing cryogenic X‐ray structures, solid‐state NMR on micro‐crystalline protein at room temperature, and solution NMR structure and dynamics, supported by MD simulations, we show that under physiologically relevant conditions the pocket is in fact shaped by pronounced open/close conformational‐exchange dynamics. The study, which is of general significance for pharmacological research, evinces a generic pitfall in drug discovery routines.

## Graphical abstract

Human carbonic anhydrase II (hCAII) plays an important role as a rigid model protein in medicinal chemistry. Under physiological conditions in solution, NMR spectroscopy combined with molecular dynamics simulations demonstrates that the active site shows pronounced open/close conformational exchange, undetected in crystallography‐based work.

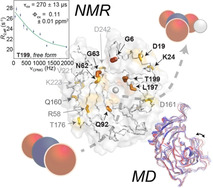

Drug discovery, the basis for successful disease treatment in our society and a multi‐trillion dollar business, relies on screening campaigns in combination with medicinal chemistry to improve binders with respect to their pharmacological properties. Besides the affinity and specificity to the target, tweaking lead compounds with respect to binding kinetics is important to endow them with appropriate efficacies and tolerable toxicity properties under physiological conditions.^[4]^ For example, fast *k*_on_ and slow *k*_off_ can be desired for drugs with intrinsic toxicity due to off‐target effects.[^4b^, ^5^] Structure elucidation for site‐specific chemical optimization of ligand properties, however, tends to rely largely on crystallographic assessment, which serves as a basis for interpreting and forecasting the interactions between proteins, ligands, and water. Methods development in medicinal chemistry, for optimizing enthalpy and entropy of binding, and for understanding and systematically improving binding kinetics, has employed well‐understood model proteins like human carbonic anhydrase II (hCAII).^[6]^ CAs are able to catalyze rapid interconversion between CO_2_ and HCO_3_^−^ and thus play an important role in almost all living organisms and tissues. Innumerable structural studies, mainly via X‐ray,^[7]^ neutron diffraction,[^1^, ^8^] and MD simulations,^[9]^ have been pursued, now providing generally applicable tools for elucidation of drug–protein and water–protein interactions. hCAII has gained its fame as a well‐known textbook example for a target generally accepted to be absolutely rigid.^[10]^ (A selection of quotes is given in the SI.) As an extremely well‐studied system with an excessive range of crystallography studies (>750 structures) on native and ligand‐bound protein, apart from the H64 side chain proton shuttle, the protein has been believed to be a target with an immobile and perfectly placed active‐site geometry and derived water network as the basis for catalytic activity and druggability (Figure 1).[^10^, ^11^]

**Figure 1:**
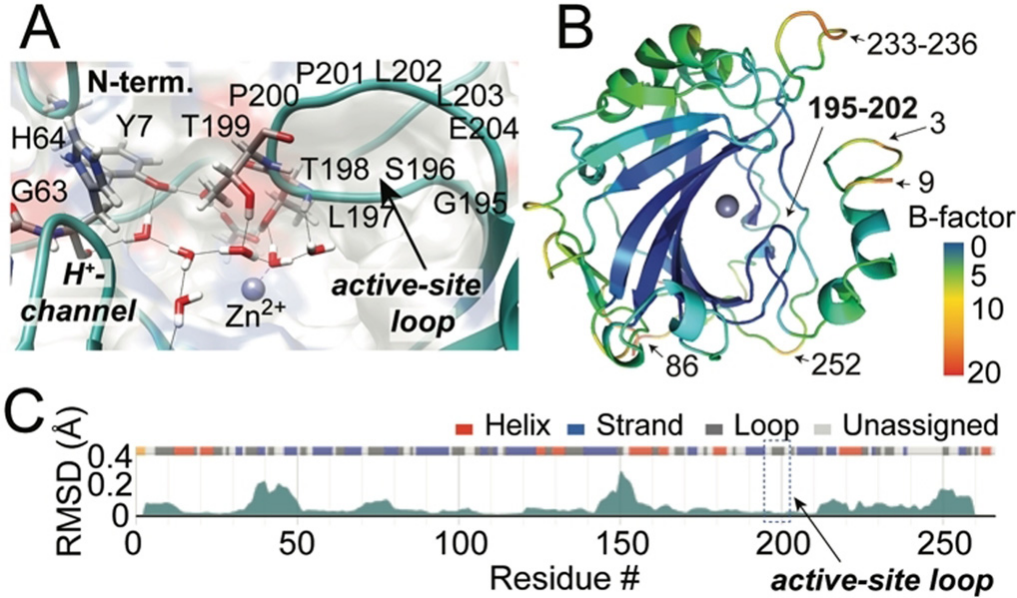
Rigidity of hCAII in previous studies. A) Conserved active site and water network spanning the active‐site loop, N‐terminal Y7, and proton shuttle H64 (PDB 4Y0J).^[1]^ B) B‐factors in PDB 2CBA. C) Structural‐conservation‐based assessment of plasticity from all X‐ray structures with >95 % sequence identity. Graphic obtained from the PDBflex server.^[3]^

Aided by solid‐state NMR studies providing complete active‐site assignments,^[12]^ we were now able to comprehensively and residue‐specifically characterize hCAII in its close‐to physiological form (monomeric in solution, at pH 7.4 and body temperature). See the SI for spectroscopic and preparative details; chemical shift assignments are listed in the BMRB under accession code 34308. In addition, we can compare the solution conditions with the assessment of the enzyme in a crystalline lattice at room temperature via solid‐state NMR at fast magic‐angle spinning (MAS),[^12b^, ^12c^] iteratively closing the gap between the cryogenic X‐ray structures (also in a crystal lattice) and monomeric, solution state conditions. Figure 2 A shows a solution NMR H/N‐HSQC spectrum, overlaid with a proton‐detected solid‐state H/N correlation of hCAII in micro‐crystals. Figure S3 shows residue‐resolved shift differences, a correlation between solution and crystal shifts, and deviating residues (Δδ>0.25 ppm) highlighted on the crystal structure (PDB 2CBA). Most importantly, T198 (compare Figure 1) is visible in solids but is exchange‐broadened in solution at the same temperature.

**Figure 2:**
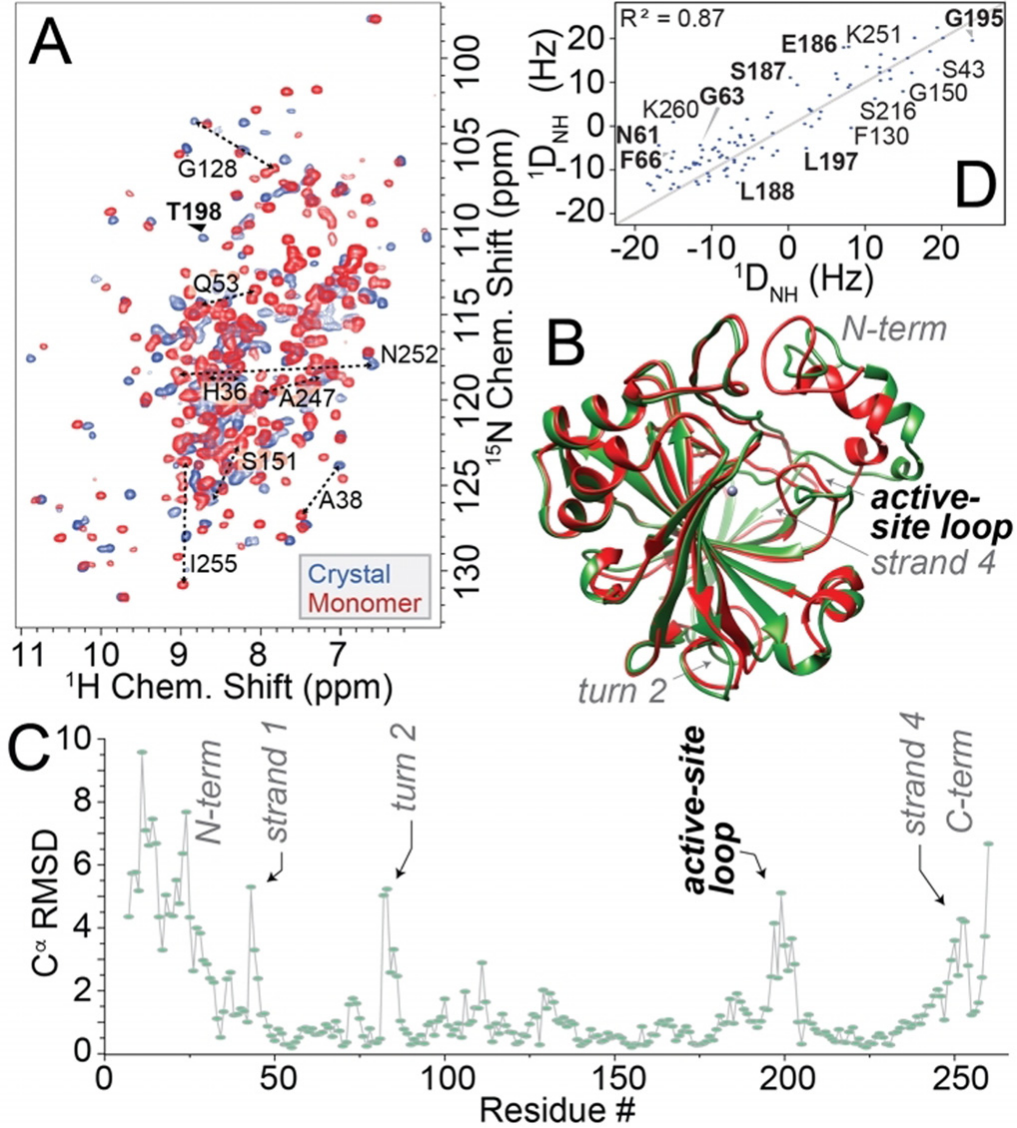
Comparison of crystalline and monomeric hCAII with respect to chemical shifts and protein structure. A) ^1^H/^15^N spectra of monomer (red) and crystal (blue) at 25 °C, also compare Figures S3–5. T198 is exchange‐broadened in solution NMR. (This is equally true for in‐cell NMR conditions, where T198 seems to be missing in HSQC spectra.^[2]^) Solid‐state spectra recorded on the same spectrometer on a uniformly ^13^C, ^15^N‐labeled microcrystalline sample of hCAII at 111 kHz MAS and similar temperature. B) Superposition of the structure in the crystalline state (PDB 2CBA, depicted in red) with the minimum‐energy solution structure (green). Strongly deviating regions are denoted. C) C^α^ RMSD between solution structure and crystalline form as a function of residue, active‐site residues in bold. D) Correlation of experimental RDCs in solution with back‐calculated RDCs based on X‐ray structure 2CBA, with a comparably poor correlation (most strongly deviating residues annotated, active‐site residues in bold).

Next, we determined the protein structure under solution conditions (Figure 2; see details in the SI text, Tables S1–3, and Figures S6–8). The atomic coordinates of hCAII were deposited in the PDB (PDB 6HD2). Expectedly, the structural organization observed in the three‐dimensional fold of hCAII in solution is overall very similar to the structure determined by crystallography (see Figure 2 B), with an RMSD (NMR vs. X‐ray) of each secondary structural element of around 0.2 to 0.7 Å, with minor differences with respect to the crystallographic structure (Figure 2 B,C) in regions that bear crystal–crystal contacts. The N‐terminus unfortunately is poorly defined due to a low number of distance restraints (see residue‐resolved precision in Figure S7). However, for the active‐site loop around the door‐keeper residue T198, with reasonable structural precision, the RMSD with respect to the X‐ray structure is high (see Figures 2 B and C). The differential placement of the active‐site loop in the solution structure has a substantial impact on the average active‐site geometry (Figure S9). To verify the above structural differences between crystalline and monomeric solution state, we also subjected the static X‐ray structure (2CBA) to the program PALES,^[13]^ generating back‐calculated residual dipolar couplings (RDCs) as expected if the H−N bond vector orientations of the crystalline structure were representative for solution conditions (Figure 2 D). Indeed, the correlation coefficient with regard to the experimentally measured RDCs in solution is only 0.87, with residues in the active site (e.g., G63, G195, L197) being among the most deviating ones, confirming a differential, slightly more open average structure under solution conditions in the absence of a crystal lattice.

Elucidating the nature of the pocket's protein–water network by solid‐state NMR, we have previously seen subtle μs timescale relaxation dispersion (RD) with unclear origin widely spread over the pocket for crystalline hCAII at room temperature.[^12b^, ^12c^] Whereas all peaks from the active‐site pocket are nicely behaved in solid‐state NMR at room temperature (compare Figure 2 A), excluding larger‐scale dynamics in the wedging crystalline lattice, in particular the catalytically important residue T198 is completely exchange‐broadened in solution at 25 °C, denoting pronounced dynamics in the active site. Hence, dynamics appear under native conditions for which timescales are slightly shifted in the presence of a crystal lattice. Increasing the temperature to 37 °C and 45 °C, we were able to undertake a detailed assessment of the physiological active‐site dynamics (see all relaxation data in Figures 3, S10–14, and S16–18). Most interestingly, slow motion on the μs timescale for the unliganded protein with *R*_2_ rates still elevated up to 37 s^−1^ (see Figures 3 A and S10) and strong RD (Figures 3 B–D and S11,12) were found locally for residues in the active‐site loop. Whereas the backbone conformational exchange we observe on the same timescale for the residues around H64, thought to exert proton shuttling via its side‐chain rotation,^[7]^ could be reconciled with the established mechanistic picture, we find the strongest RD and highest *R*_2_ rates at the very bottom of the active site (Figure 3 B–D). The conformational exchange can be fitted individually (Figure 3 C/D) or collectively over the active site (Figure S11) and involves the whole active‐site loop from S196 to E204 (see Figures S11 and S12 for the dispersion curves and peak shapes at 45 °C and 37 °C, respectively). Fitting the RD profiles within the loop globally yields an exchange lifetime of 270 μs, for which exchange contributions *R*_ex_ are depicted by differential coloring in Figure 3 C. Interestingly, residues G63 (H64 is unfortunately overlapped), which site is coupled to the active‐site loop through the H‐bond network, and G6 at the very N‐terminus show strong RD on the same timescale and could be included in the global fit (Figures 3 B/D and S14).

**Figure 3:**
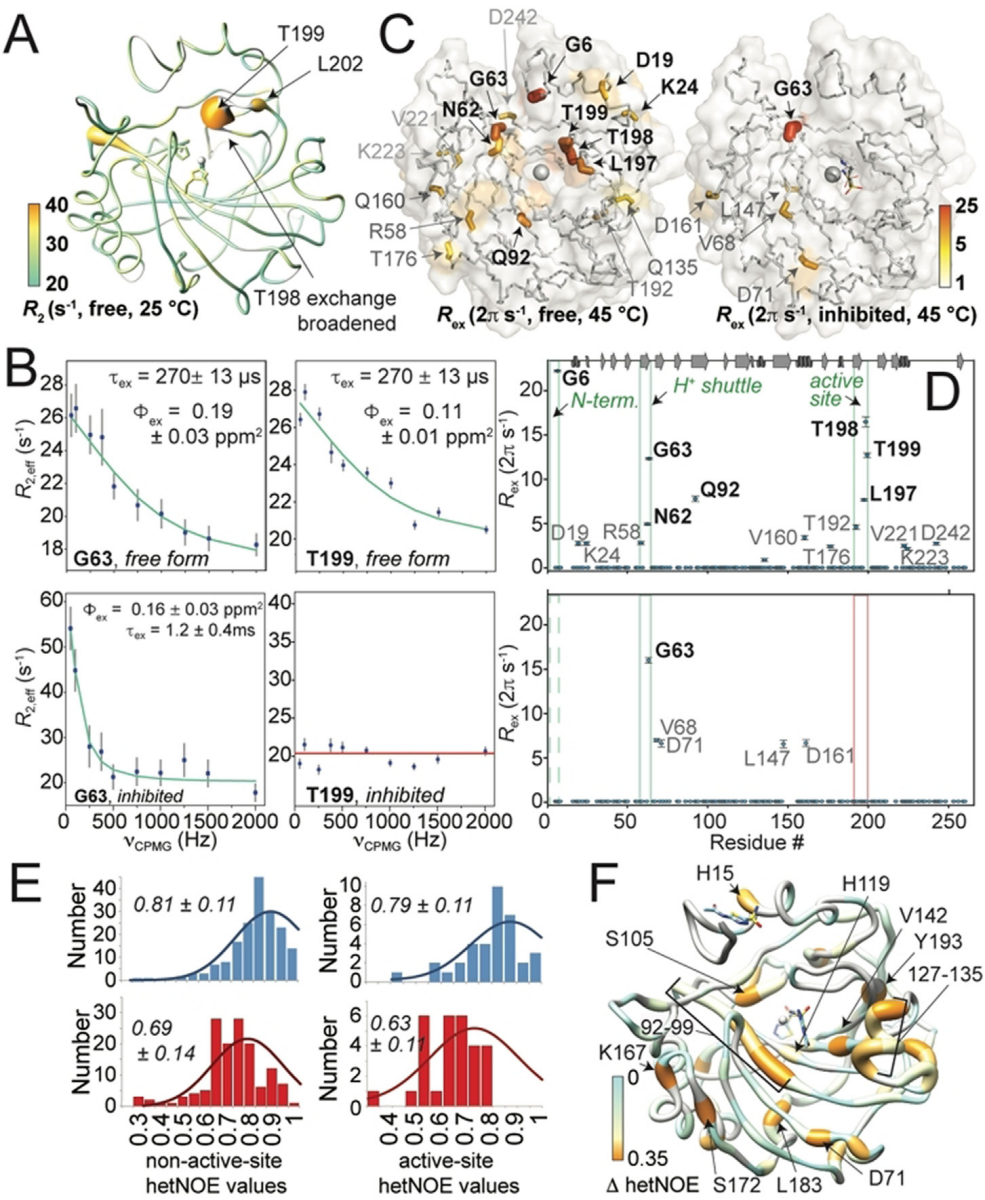
Dynamics of physiological hCAII in solution. A) *R*_2_ rates exceeding 20 Hz (cyan, as expected for a globular 29 kDa protein without conformational exchange). B) RD profiles from a collective fit of the outer pocket close to H64 and active‐site loop in the absence (upper row) and presence of inhibitor (lower row). C) Exchange contribution from RD in the absence (left) and presence of inhibitor (right) depicted on the structure. D) Residues with significant exchange contributions in the absence (upper row) and presence (lower row) of inhibitor as a function of sequence. G6 has too low signal‐to‐noise ratio for quantitative fitting and therefore is not shown in the plot. Data in (C) and (D) represent individual fits. E) Shift of hetNOE distributions (histograms and Gaussians fits as well as raw‐data mean and standard deviation) for non‐active‐site (left) and active‐site residues (right) of ligand‐bound (red) and non‐liganded protein (blue). F) Site‐specific decrease in hetNOE of liganded with respect to free form.

Next, in order to mechanistically assess the nature of the active‐site conformational exchange in more detail, we carried out molecular dynamics (MD) simulations with Gromacs^[14]^ (see the SI for details). Dynamics on the 100 μs timescale are very challenging to capture in MD, which would unavoidably have obscured motion on this timescale also in previous studies. The active‐site loop is packed against the N‐terminal part of hCAII via adjacent hydrophobic surfaces. Whereas these contacts would not abolish loop motion, they are expected to slow down the dynamics of the active site loop. Previously, N‐terminal truncation of hCAII up to residue 24 was shown to largely retain catalytic activity, with a penalty of around 1 kcal mol^−1^ on the activation energy for catalysis and a remaining 10^5^ turnovers per second.^[15]^ In addition to the intended acceleration of the active‐site loop dynamics, coupling between the N‐terminus and the active site, which is also evident from our RD data above, will naturally remain elusive in MD simulations using such an N‐terminally truncated protein. However, these simulations can grasp the different intrinsic interactions and plasticity of the active site of unliganded vs. inhibitor‐bound hCAII and unravel the mechanics underlying the experimental observations. Indeed, without the increased barrier due to the N‐terminal interactions, the dynamics of the active‐site loop is witnessed in MD within a 500 ns timescale. In the absence of an inhibitor, the loop is found to easily and reversibly detach from the catalytic center in a collective hinge motion by unlatching its H‐bonds to the Zn‐bound hydroxide and E106 sidechain, with the tip of the loop around T198 showing the largest displacement. Figure 4 A–C shows MD simulations of the unliganded protein as well as with the inhibitor dorzolamide, in which this pronounced plasticity of the active‐site loop is not observed (see below).

**Figure 4:**
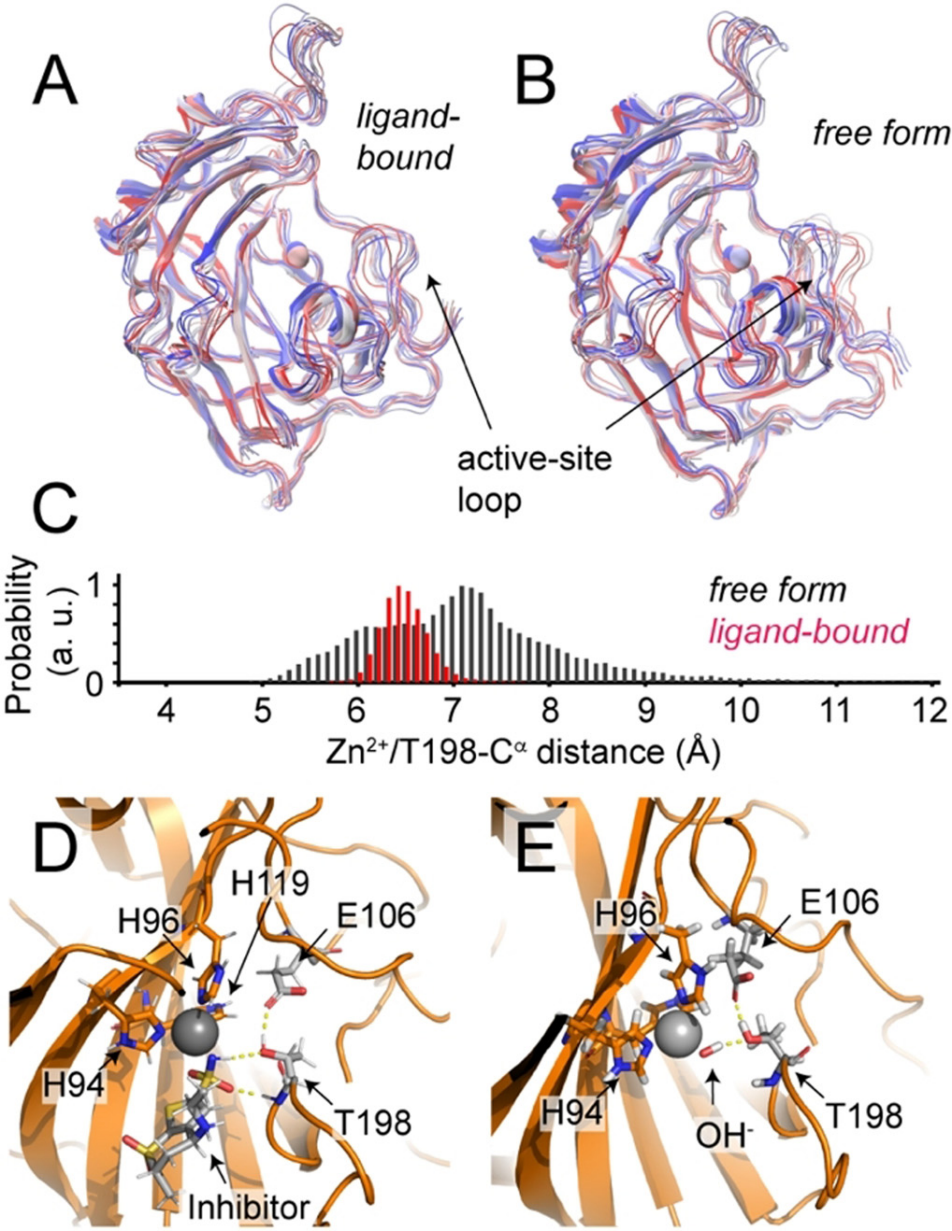
Mechanical assessment of loop interactions using MD simulation of N‐terminally truncated hCAII. A) In the ligand‐bound form, the active‐site loop is locked in its closed position. B) Without inhibitor, the loop shows pronounced open/close dynamics. C) Histogram of Zn–T198 distance in the presence (red) and absence (black) of inhibitor. D) Stabilization of the closed loop conformation by multiple H‐bonds to the inhibitor. E) Zn–OH^−^‐based H‐bonds of the active‐site loop in the absence of inhibitor.

Drug discovery on CAs has afforded manifold sulfonamide inhibitors, which are known to replace the Zn‐bound catalytic water molecule. Hence, they also impair the conserved water network, one of the features of the CA core.[^1^, ^16^] Changes in active‐site B‐factors induced by anti‐glaucoma drugs like acetazolamide or dorzolamide are insignificant in the crystal, and structures are virtually identical to the inhibitor‐free form (see Figure S15).^[17]^ This has led to the conclusion that neither structural nor entropic changes are associated with inhibitor binding in CAs and that contributions to the binding kinetics only stem from the drug itself as well as replaced water molecules.^[6]^ The application of the NMR methods described above to a dorzolamide‐inhibited hCAII, however, as shown in Figure 3 B–D, tells a different story. Upon binding of the inhibitor, the observed motion of the active‐site loop ceases, imposing an entropy change compared to the free enzyme (also compare Figures S16 and S17). This reduced motion is observed consistently in the experimental data as well as in the MD simulations of the N‐terminally truncated protein (Figure 4 A/C). By contrast, G63 conformational exchange is not quenched. However, it is slowed down to the ms regime in the presence of the inhibitor. The N‐terminal residue G6 in the ligand‐bound state yields a very low signal‐to‐noise ratio, rendering its RD profile ambiguous. In addition, changes regarding ps‐ns timescale motion upon ligand binding can be deduced from *R*_1_ and hetNOE data. In the uninhibited case, fast‐timescale motion is present only at the tips of some external loops, which are associated with structural deviations to the crystalline state (see above). hetNOE values in the absence of inhibitor scatter around 0.8 for the whole sequence, again showing slightly elevated fast‐timescale motion for the loop around T198. (hetNOE values are also part of Figure S10 and S16.) By contrast, upon inhibition, fast‐timescale motion (decreased hetNOE values) is observed for residues in large parts of the primary sequence. Statistics and differences, liganded vs. unliganded form, are shown in Figure 3 E and plotted on the protein structure in Figure 3 F, respectively. All statistics are shown in Figure S17. Figure S18 also shows the effect of CO_2_ binding to the active site as the natural substrate. Its affinity is approximately 100x lower than the inhibitor, such that these data only show subtle effects. The trends, however, seem to be in line with the sulfonamide as a high‐affinity (covalent) substrate analogue.

Our various observations unambiguously demonstrate the existence of conformational‐exchange backbone dynamics in the active site of hCAII under close‐to physiological conditions. This contradicts the conclusions from X‐ray crystallography‐based studies[^17^, ^18^] that have made the enzyme the drug discovery textbook example for a highly rigid drug target. The presence of strong, spontaneous conformational exchange in the active site of CAs on one hand challenges the mechanistic model of a highly rigid active‐site reaction chamber for catalysis. On the other hand, as a consequence, the use of the target for a systematic understanding of binding kinetics and underlying entropic features, as well as for designing methodology for lead optimization, has been overlooking a decisive property of the pocket over decades. Whereas the loop remains in the closed position shown in the X‐ray structures when a high‐affinity inhibitor providing multiple H‐bonds is bound in the active site, the weak H‐bonding between T198 and the Zn‐bound catalytic water/OH^−^ in the inhibitor‐free form (Figure 4 D/E) is easily opened at room temperature in the absence of a crystal lattice, such that the active‐site loop undergoes pronounced open/close dynamics. The timescale of the active‐site loop motion is further modulated by the hydrophobic interactions with the N‐terminus, which couples the dynamics of these two structural regions. As such, the protein has the possibility to dynamically adjust the active site towards a suitable geometry. The conformational‐exchange timescales are comparable to the catalytic turnover rate of the enzyme, underlining its likely relevance for biological activity. The sampling of open and closed conformations in the absence of a substrate may be advantageous for substrate intake and also adds to understanding hCAII substrate flexibility.^[19]^ In addition, the results for the substrate analogue inhibitor and trends for bicarbonate as a substrate suggest that such conformational changes of this region may be switched off in the event of substrate binding, where the increased fast‐timescale motion observed might be beneficial for conversion and product release instead. Apart from the unveiled entropic contributions important for binding kinetics and affinities, the conversion of local slow‐timescale motion into fast‐timescale fluctuations in large parts of the protein upon accommodation of an active‐site inhibitor also suggests coupling of active‐site plasticity with the overall protein architecture. Whereas a crystal lattice seems to modulate the extent and timescale of dynamics merely on a quantitative level,^[12c]^ cryogenic temperature as well as the insensitivity of crystallography to low excited‐state populations bear the risk of drawing a misleading picture of a target's personality in standard drug discovery pipelines.

Here, we have demonstrated important differences for properties in the active site of hCAII, unmatched “rigid” model system for drug discovery, under biologically representative conditions in solution compared to previous crystallography‐based studies. Using NMR relaxation, relaxation dispersion, RDCs, and MD simulations, we have demonstrated that μs timescale backbone conformational exchange between open and closed forms exists for the important active‐site loop under native conditions. The active‐site plasticity hitherto undetected for this prominent target calls for awareness upon assessing enthalpy and entropy of binding and in structure‐guided lead optimization via standard approaches.

## Conflict of interest

The authors declare no conflict of interest.

## Supporting information

As a service to our authors and readers, this journal provides supporting information supplied by the authors. Such materials are peer reviewed and may be re‐organized for online delivery, but are not copy‐edited or typeset. Technical support issues arising from supporting information (other than missing files) should be addressed to the authors.

Supplementary
