# Supplementary material for: The Active Site of a Prototypical “Rigid” Drug Target is Marked by Extensive Conformational Dynamics
Source: Angew Chem Int Ed Engl. 2020 Nov 16;59(51):22916–21. doi: 10.1002/anie.202009348 (PMC7756556; doi:10.1002/anie.202009348)
Supplement: Supplementary file 1 — Supplementary [file ANIE-59-22916-s001.pdf]

Angewandte Chemie
Eine Zeitschrift der Gesellschaft Deutscher Chemiker

Supporting Information

The Active Site of a Prototypical "Rigid" Drug Target is Marked by Extensive Conformational Dynamics

*Himanshu Singh, Chandan K. Das, Suresh K. Vasa, Kristof Grohe, Lars V. Schäfer, and Rasmus Linser**

anie_202009348_sm_miscellaneous_information.pdf

Table of Contents

Exemplary quotes evincing the accepted picture of CAs as rigid enzymes	S3
Materials and Methods	S3
Fig. S1: Size-exclusion profile	S7
Fig. S2: Comparison of HSQC spectra	S7
Fig. S3: Chemical shift differences between solution and crystalline state	S8
Fig. S4: Overlay of HSQC spectra for hCAII and hCAII:acetazolamide complex	S8
Fig. S5: Residue-specific chemical shift perturbations	S9
Fig. S6: Chemical shift index	S9
Table S1: Experimental RDCs	S10
Table S2: H-bond restraints	S11
Table S3: NMR structural statistics	S12
Fig. S7: Residue-resolved RMSD	S13
Fig. S8: hCAII monomeric structure in solution	S13
Fig. S9: Comparison of active-site-loop conformation between NMR and X-ray	S13
Fig. S10: ^{15}N relaxation non-ligand-bound vs. acetazolamide-bound hCAII	S14
Fig. S11/12: Relaxation dispersion profiles at 45 °C and at 37 °C	S15
Fig. S13: Solid-state NMR relaxation dispersion of the active-site loop	S16
Fig. S14: Comparison of G6 dispersion profiles	S16
Fig. S15: Comparison of non-liganded and dorzolamide-bound X-ray structures	S17
Fig. S16: Sausage representation of R_2 difference values	S17
Fig. S17: Distributions of relaxation values	S17
Fig. S18: ^{15}N relaxation of non-ligand-bound vs. bicarbonate-bound hCAII	S18
Assessment of protection of active-site regions of hCAII against H/D exchange	S18
Fig. S19: hCAII active-site HD exchange in solution	S19
Table S4: Exponentially fitted HDX rates	S20
Fig. S20: Faster H/D-exchange	S23
Fig. S21: Strong H-bonds depicted on the crystal structure	S23
Fig. S22: Exchange-hindered residues	S24
References	S24

Exemplary quotes evincing the accepted picture of CAs as rigid enzymes and drug targets:

Titel of chapter 8.2.2.1 in „Small Molecule — Protein Interactions“ (Springer, 2003): „*8.2.2.1 Carbonic Anhydrase as an Example for a Rigid Enzyme*”

Alterio et al., Org. Biomol. Chem. 8 (15), 3528-3533 (2010): “*The CA active site (of all α-CA isozymes crystallized so far) possesses two distinctive features: ... (ii) a rigid 3D structure of the protein backbone. Indeed, no modification of the protein backbone has been reported when inhibitors, activators and substrates bind to the enzyme cavity.*”

Abstract of Snyder et al., Proc. Natl. Acad. Sci. U. S. A., 108, 17889–17894 (2011) on hCAII: “*In binding to this structurally rigid protein, a set of ligands (also defined to be structurally rigid) shows the expected gain in binding free energy as hydrophobic surface area is added.*”

A. Liljas, IUCrJ, 5, 4-5 (2018): “*The high rate of the enzyme is evidently due to the weak binding of the substrates, as well as the firm structure of its active site, the only moving part being His64.*”

…

Materials and Methods

Overexpression and purification of hCAII: The cDNA encoding *hCAII* was cloned into a pGEX expression vector and transformed into *E. coli* strain BL21(DE3) codon plus cells for over-expression, isolation and purification of the protein as described earlier.[1] A representative size-exclusion chromatography profile of purified (protonated $^{13}C/^{15}N$) hCAII is shown in Fig. S1. Both, the size exclusion profile (Fig. S1) as well as the rotational correlation time of 11 ns determined from bulk R_1 and R_2 rates denote the protein to be monomeric under the conditions of the solution NMR measurements.

NMR Spectroscopy: For NMR studies, uniformly ^{15}N-labelled (u-^{15}N), $^{13}C/^{15}N$-doubly-labelled (u-$^{13}C/^{15}N$), and uniformly $^{2}H/^{13}C/^{15}N$-triple-labeled (u-$^{2}H/^{13}C/^{15}N$) hCAII were prepared in a mixed solvent of 90% H_2O and 10% $^{2}H_2O$ (50 mM sodium phosphate, 50 mM NaCl). CAII is found mostly in human erythrocytes (pH 7.4 and 37 ºC). We thus performed all solution NMR studies at pH 7.4 and between 25 and 45 ºC, which should thus represent close-to-physiological conditions. Whereas this represents the closest to physiological we can get to when high signal-to-noise ratio is required, in-cell NMR spectra of hCAII have been obtained recently.[2] The study confirms that hCAII is free from severe interactions with slow-tumbling cellular components, which would otherwise circumvent the well-behaved spectra obtained, and both T198 and the sidechain amide of N67 (part of the active-site water network[3]) are exchange-broadened just like under *in-vitro* conditions, reflecting the representativeness of the results presented here also for the cellular case. pH and temperature are also very similar to the previous solid-state NMR (ssNMR) studies alluded to in the main,[1, 3-4] and ssNMR conditions

in micro-crystals again are very similar to the conditions of previous crystallographic studies (apart from the temperature). Both reflects protein in a crystalline lattice (opposed to monomeric conditions in solution), again without any obvious, specific dimer formation. All triple resonance NMR experiments were carried out at 25 ºC with protein concentrations between 0.5 to 0.6 mM on a Bruker Avance 800 MHz NMR spectrometer equipped with a 5 mm cryogenically cooled triple-resonance probe and a pulsed-field gradient. A suite of 3D double- and triple-resonance NMR experiments were performed for sequence-specific ^{1}H, ^{13}C and ^{15}N backbone resonance assignments as discussed earlier.[5] In addition, we recorded 3D experiments such as HCCH-TOCSY (mixing time = 24 ms), [^{15}N, ^{1}H]-NOESY-HSQC (mixing time = 100 ms) and [^{13}C, ^{1}H]-NOESY-HSQC (mixing time = 80 ms) for almost complete assignment of ^{1}H, ^{13}C and ^{15}N side-chain resonances and for the determination of NOE distance constrains used in the 3D structure calculation of the enzyme. ^{1}H/^{15}N-RDCs were collected using Pf1 filamentous phage (purchased from Asla Biotech) with a concentration of 15 mg/mL as an alignment medium, resulting in $^{1}H^{N}$-$^{15}N^{H}$ residual dipolar couplings between -15 and +18 Hz. The RDCs were determined via IPAP HSQC experiments.[6] Alignments of the hCAII sample was confirmed by a D_2O quadrupole splitting of 14 Hz. PALES[7] was used to calculate the alignment tensor, resulting in a tensor magnitude of 13.91 Hz and a rhombicity of 0.252. The complete backbone and sidechain ^{1}H, ^{13}C and ^{15}N resonance assignments of hCAII were deposited into the BMRB (http://www.bmrb.wisc.edu) under the accession number 34308. The assignments of backbone amide ^{1}H and ^{15}N chemical shifts of hCAII in complex with the sulfonamide inhibitor acetazolamide were obtained by comparison to the assignments of free hCAII and confirmation via 3D ^{15}N-edited NOESY and TOCSY experiments. The ^{1}H chemical shifts were referenced with respect to the external standard 2,2-dimethyl-2-silapentene-5-sulfonates (DSS), while the ^{13}C and ^{15}N chemical shifts were referenced indirectly.

NMR Relaxation: The backbone ^{15}N T_1 relaxation measurements at 800 MHz were acquired using recovery delays of 10, 50, 100, 200, 300, 500 and 700 ms. The ^{15}N T_2 measurements were carried out with the same acquisition parameters using a CPMG pulse sequence[8] with relaxation delays of 0.5, 1, 5, 10, 20, 30, and 40 ms. Steady-state [^{15}N, ^{1}H] heteronuclear-NOE measurements were carried out with and without proton saturation during the relaxation delay. In these NOE-experiments, 5 s of relaxation delay and 3 s of proton saturation (or 8 s of relaxation delay only) were used. The heteronuclear-NOE values were determined as the ratio of the peak intensities measured from the spectra acquired with and without proton saturation. NMR spectra were processed using TopSpin3.5 (Bruker BioSpin) and analyzed using CARA[9] and CCPN[10].

Constant-time ^{15}N-CPMG (CT-CPMG) relaxation dispersion experiments[11] were measured at 25, 37, and 45 °C. Experiments were performed with a constant-time delay of 40 ms, and 9 variable CPMG frequencies (ν_{CPMG}) ranging from 50 to 2000 Hz were collected. Besides, for each data set the frequencies 700 and 150 Hz were repeated for estimation of error in $R_{2,eff}$ and a reference spectrum without constant time delay (T_{CPMG} = 0) was recorded. For each 2D dataset corresponding to one CPMG frequency, 128 and 2048 complex points in the indirect and direct dimensions, respectively, were collected with 32 scans. 2D datasets for all frequencies were

measured in a scan-interleaved fashion. The recycle delay of 1.5 s was used, giving rise to a net acquisition time of approximately 2.6 h per data set.

NMR relaxation data were processed using Topspin 4.0.8. Peak intensities were quantified and visualized by using SPARKY[12]. The effective amide ^{15}N transverse relaxation rate at each CPMG frequency was calculated according to the relation of effective transverse relaxation rate ($R_{2,\,eff}$),

$$R_{2,eff} = \frac{-\ln\left(\frac{I}{I_0}\right)}{T}, \quad (1)$$

where I is the peak intensity, Io is the corresponding intensity in a reference spectrum recorded without the CT-CPMG relaxation period and T is a constant time delay. Data were analyzed individually using the NESSY software package[13] to obtain the kinetic parameters of interest, corresponding to a two-site exchange process. NESSY fits the profiles to no-exchange and fast-exchange mathematical models. It then chooses the best model for each residue based on the corrected Akaike information criterion.

Model 1: no exchange

$$R_{2,\,eff} = R_2^0 \quad (2)$$

Model 2: two states, fast exchange

$$R_{2,\,eff} = R_2^0 + \phi_{ex}/k_{ex}[1.0 - (4\nu_{CPMG}/k_{ex})*\tanh(k_{ex}/4\nu_{CPMG})] \quad (3)$$

For global analysis, a two-state model (*i.e.* with the same kinetic parameters for all dispersion profiles considered) was fitted to the data using the program SHEREKHAN[14], which minimizes the target function by numerically propagating mathematical equations such as the Bloch-McConnell, Carver-Richards and Luz-Meiboom models.

Solid-state ^{15}N $R_{1\rho}$ measurements were recorded as pseudo-3D, proton-detected HN correlation spectra and data fitting was done as described previously.[3]

NMR Structure Calculation: The 3D solution structure of hCAII was determined using the following NMR constraints: (i) Dihedral angle constraints derived using TALOS-N[15] with the knowledge of individual $^1H^N$, ^{15}N, $^{13}C\alpha$, $^{13}C\beta$, ^{13}CO chemical shift values as inputs. A total of 387 ϕ and ψ dihedral angle constraints were used. (ii) Generic hydrogen bond (H-bond) constraints were imposed for residues located at well-defined α-helical and β-strand regions. In addition, H-bond restraints originating from H/D-exchange studies (indicating presence of a strong H-bond in solution) were used as shown in Table S3. (H-bond acceptors were defined as found in crystals, using pdb 2cba and HBOND, cib.cf.ocha.ac.jp/bitool/HBOND.) In all cases, an upper limit of 2.4 Å was used for the H-O distance. A total of 156 H-bond constraints were used. (iii) Cross peaks in NOESY spectra were identified and automatically assigned using ARIA 2.3[16]. The upper-bound distance constraints were set to 6.0 Å, respectively. An initial extended model consisting of a Zn^{2+} ion, tetrahedrally coordinated with H94, H96, H119 residues and a water molecule, was created using the CNS program[17]. A total of 3892 distance constraints, which included 764 intra-residue, 1098 inter-residue (sequential), 676 medium-range, and 1354 long-range distance constraints, were used in the 3D structure calculation. With all these restraints as input, amounting to ~17 restraints/residue, the 3D structure of hCAII was calculated using the simulated annealing protocol in ARIA 2.3. A total of 500 structures were

calculated, from which 20 structures with lowest target function and no distance or angle violations were selected. These 20 conformers with lowest target function were further refined in explicit water with 115 $^{1}H/^{15}N$-RDC restraints, NMR-derived distance restraints and angle restraints using the inbuilt ARIA 2.3 CNS program. A correlation of 0.97 between back-calculated and experimental RDCs (see Fig. S8B) indicates good agreement between observed data and calculated structure. The program PSVS-1.4 (http://www.psvs-1_4.nesg.org) was used to validate the quality of the selected ensemble of lowest-energy structures of hCAII. The 3D coordinates of individual atoms of hCAII thus obtained were deposited in the PDB (pdb id: 6HD2). The structure figures were prepared using Pymol (The PyMOL Molecular Graphics System, Version 1.8 Schrödinger, LLC) and UCSF Chimera.

H/D exchange studies: Non-equilibrium H/D-exchange was measured on u-^{15}N hCAII (lyophilized from aqueous phosphate buffer at pH 7.5 and redissolved in D_2O) by recording consecutive ^{15}N-^{1}H HSQC spectra over a period of 18 days (7 days continuous measurement acquisition time, using 15 to 45 min per spectrum). The intensity decay was fitted with a double exponential function as shown in equation 1.

$$I(t) = A_1 e^{-k_1 t} + A_2 e^{-k_2 t} \quad (1)$$

The exchange rate at physiological pH generally depends on two different properties, the H-bond opening rate and the water accessibility of the site. The opening-rate of the H-bond is represented by an exponential decay rate of the HSQC-peak intensity, while hindered water accessibility, if present, results in a disturbance of this exponential function, either making it a multi-exponential decay (fitted as double exponentially decay) or effectively resulting in no decay at all.[18]

MD simulations:
All MD simulations were carried out with Gromacs 2019.2[19]. For apo hCAII, the X-ray crystal structure 3KS3[20] was used as a starting structure, whereas the dorzolamide-bound simulations were initiated from the X-ray structure 4M2U[21]. To create the N-terminally truncated proteins, the coordinates of the first 24 residues were deleted from 3KS3 and 4M2U, respectively, i.e., the first residue in the chain is Gly25. After adding hydrogen atoms to the protein, inhibitor, and the crystal water (the protonation states of all ionizable residues were set corresponding to pH=7), the systems were solvated with about 10.000 SPC/Eb water molecules in a periodic rhombic dodecahedron box. The Amber ff15ipq[22] protein force field was used. For the coordination of the Zn ion, we followed the metal center parametrization protocol of Merz and coworkers[23], using the MCPB.py program as available in the Amber18 package. Lennard-Jones 12-6 interactions were smoothly shifted to zero at a 1.0 nm cut-off; this distance was also used for switching between short- and long-range electrostatic interactions, which were treated with the particle mesh Ewald algorithm[24]. After energy minimization, the systems were heated up to 300 K during 200 ps in the NVT ensemble; in these equilibration simulations harmonic position restraints with force constants of 1000 kJ/mol/nm^2 were applied to all protein heavy atoms. Afterwards, two subsequent 200 ps NpT simulations with position restraints were carried out. In the first run, the position restraints were applied to all protein heavy atoms, and in the second run the position restraints on the protein side chain atoms were released. Finally,

all position restraints were switched off. Temperature and pressure were kept constant at 300 K and 1 bar using the thermostat of Bussi and coworkers[25] and the Berendsen barostat, respectively. The SETTLE and LINCS constraint algorithms were applied to constrain internal degrees of freedom of the water molecules and all protein bonds with H-atoms, respectively, allowing to integrate the equations of motion with 2 fs time steps. Finally, for both apo and dorzolamide-bound hCAII, 20 independent 500 ns MD simulations were initiated using different random seeds for the atomic velocities drawn from a Maxwell-Boltzmann distribution at 300 K. These sets of simulations were carried out for both the full-length proteins as well as the N-terminally truncated ones.

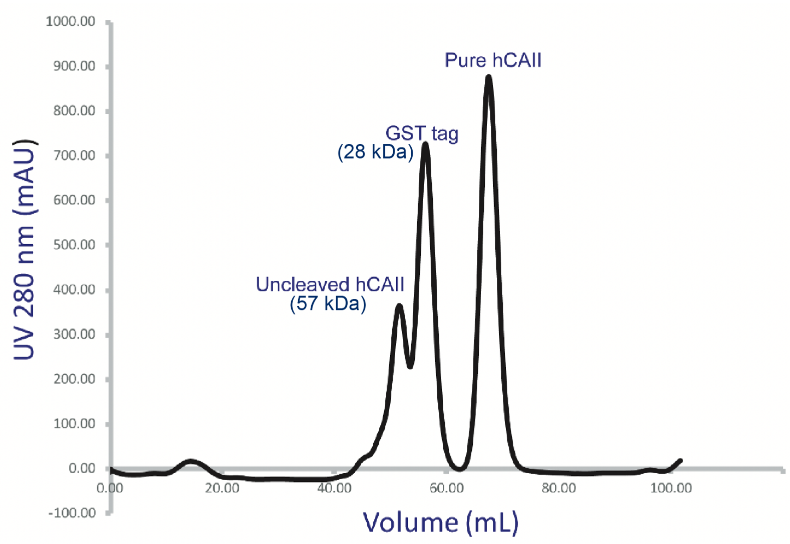

Fig. S1: Size-exclusion chromatography profile of purified hCAII.

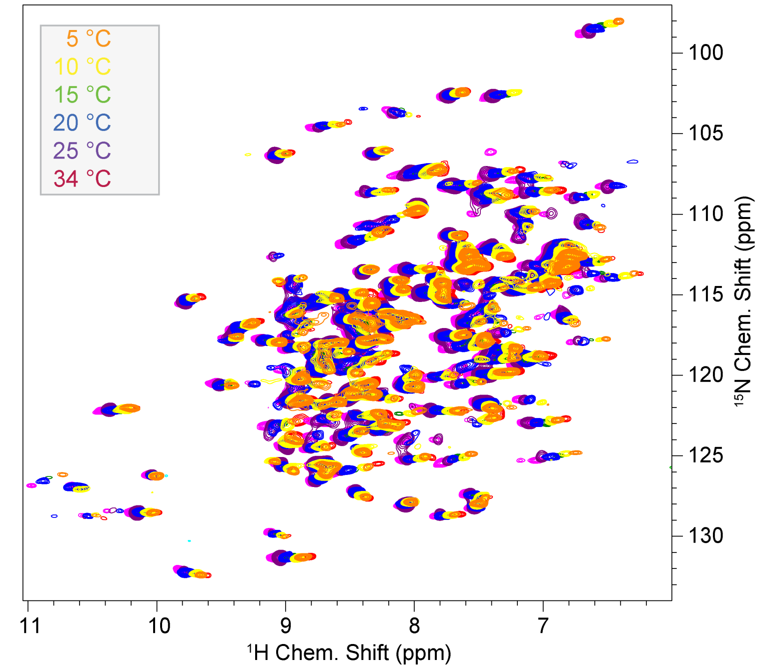

Fig. S2: Comparison of hCAII solution [^{1}H,^{15}N]-HSQC spectra under various temperatures.

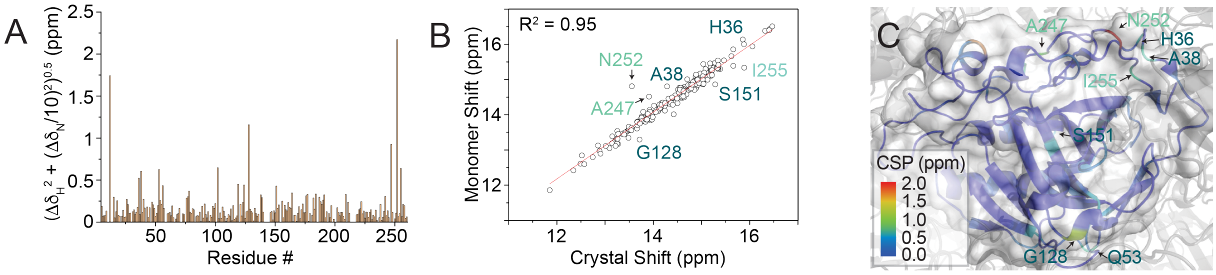

Fig. S3: Chemical shift differences between solution hCAII and hCAII in the crystalline state[1]. **A)** CSPs as a function of residue. **B)** Correlation plot of chemical shifts in monomeric and crystalline hCAII, with effective amide shifts $\sqrt{(\delta_H^2 + \delta_N^2/100)}$. **C)** Shift differences and their extent, color-coded by origin on pdb 2CBA, dark green representing direct changes obviously from crystal-crystal contacts and cyan such that must be indirectly affected by conformational changes. Solid-state spectra recorded on the same spectrometer on a protonated, uniformly ^{13}C, ^{15}N-labeled microcrystalline sample of hCAII at 111 kHz magic-angle spinning and similar temperature.

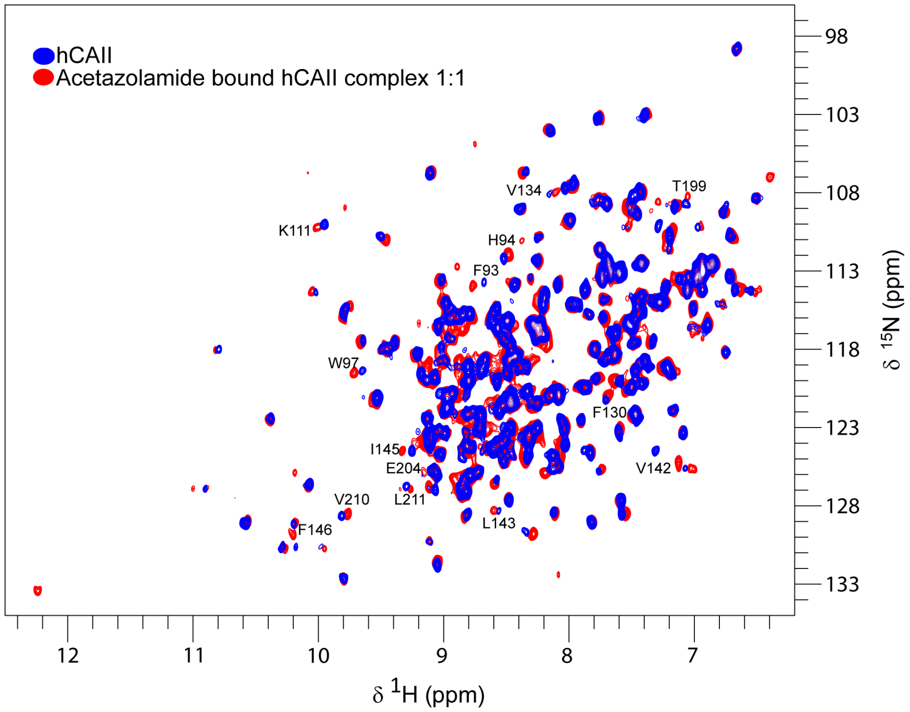

Fig. S4: Overlay of 2D [^{1}H, ^{15}N]-HSQC spectra for hCAII (blue) and hCAII:acetazolamide complex (red) under similar experimental conditions at 25 ºC.

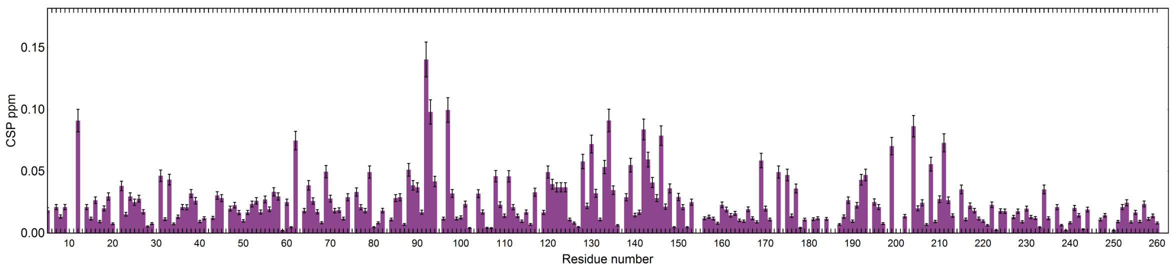

Fig. S5: Residue-wise plot of chemical shift perturbations (considering only amide proton and amide nitrogen chemical shifts) upon hCAII:acetazolamide complex formation.

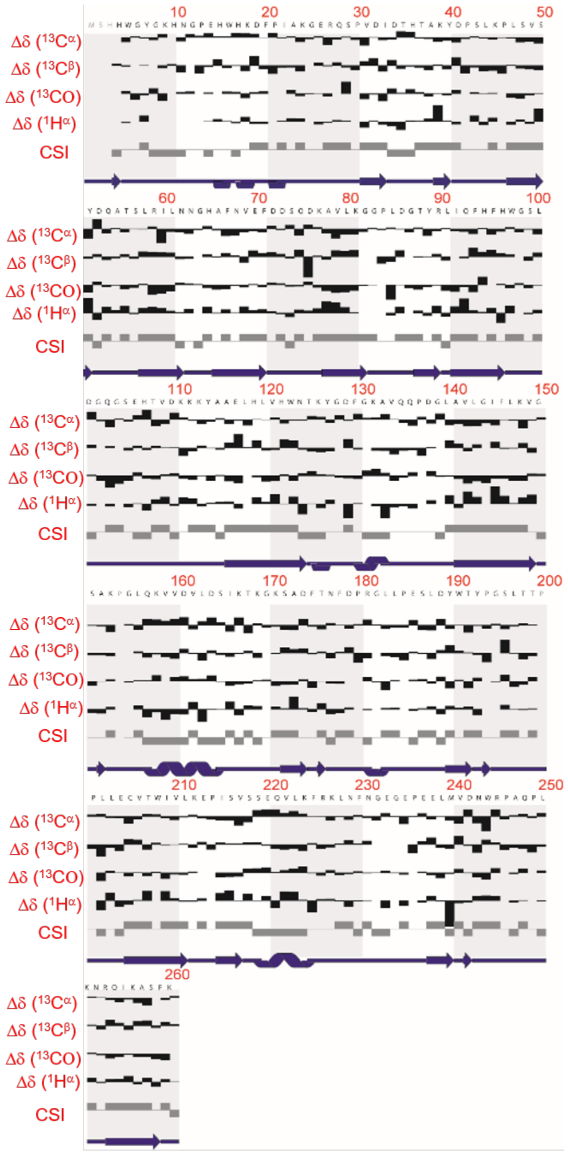

Fig. S6: Chemical shift index derived from the complete resonance assignments of hCAII.

Table S1: Experimental RDCs measured using Pf1 phages (Tensor: 1, magnitude 13.91, rhombicity 0.252).

Res.	Type	1	2	RDC		Res.	Type	1	2	RDC
7	Tyr	N	H	-8.3400		127	Tyr	N	H	2.730
8	Gly	N	H	8.070		128	Gly	N	H	-14.320
9	Lys	N	H	-10.520		129	Asp	N	H	2.140
14	Glu	N	H	-5.730		130	Phe	N	H	8.190
16	Trp	N	H	-6.970		131	Gly	N	H	13.330
17	His	N	H	13.200		135	Gln	N	H	16.450
20	Phe	N	H	18.480		136	Gln	N	H	-8.000
25	Gly	N	H	-11.200		150	Gly	N	H	14.950
27	Arg	N	H	-8.030		158	Lys	N	H	-15.340
32	Asp	N	H	-4.800		159	Val	N	H	-11.760
36	His	N	H	-8.990		163	Leu	N	H	-12.900
38	Ala	N	H	3.000		166	Ile	N	H	-2.230
39	Lys	N	H	-8.670		167	Lys	N	H	-6.220
40	Tyr	N	H	12.010		172	Ser	N	H	-7.050
41	Asp	N	H	-15.860		177	Asn	N	H	-2.720
43	Ser	N	H	19.490		178	Phe	N	H	-3.440
47	Leu	N	H	16.090		179	Asp	N	H	-4.580
48	Ser	N	H	-11.970		183	Leu	N	H	10.220
49	Val	N	H	-5.630		184	Leu	N	H	12.050
50	Ser	N	H	-10.320		186	Glu	N	H	7.870
55	Thr	N	H	-9.570		187	Ser	N	H	0.260
56	Ser	N	H	-11.350		188	Leu	N	H	-6.640
59	Ile	N	H	-13.410		189	Asp	N	H	-7.700
60	Leu	N	H	-16.760		190	Tyr	N	H	-11.990
61	Asn	N	H	-16.920		195	Gly	N	H	24.000
62	Asn	N	H	-15.370		197	Leu	N	H	2.340
63	Gly	N	H	-9.100		199	Thr	N	H	11.930
66	Phe	N	H	-15.190		202	Leu	N	H	-5.190
67	Asn	N	H	-17.060		204	Glu	N	H	-4.530
68	Val	N	H	-14.980		212	Lys	N	H	2.970
70	Phe	N	H	-6.300		213	Glu	N	H	13.840
73	Ser	N	H	-12.340		216	Ser	N	H	11.300
74	Gln	N	H	6.210		219	Ser	N	H	-9.870
81	Gly	N	H	4.240		221	Gln	N	H	-8.000
82	Gly	N	H	-8.040		222	Val	N	H	-1.440
84	Leu	N	H	-2.000		224	Lys	N	H	-10.420
86	Gly	N	H	-17.990		228	Leu	N	H	-15.000
87	Thr	N	H	5.400		231	Asn	N	H	1.150
90	Leu	N	H	-5.380		232	Gly	N	H	-2.050
92	Gln	N	H	-12.130		235	Glu	N	H	-11.560
96	His	N	H	-18.340		238	Glu	N	H	19.410
99	Ser	N	H	4.030		241	Val	N	H	-7.060
100	Leu	N	H	-8.940		243	Asn	N	H	-6.620
104	Gly	N	H	-11.500		244	Trp	N	H	11.910
112	Lys	N	H	-6.000		245	Arg	N	H	7.810
113	Lys	N	H	15.550		251	Lys	N	H	7.200
115	Ala	N	H	-13.960		254	Gln	N	H	20.170
117	Glu	N	H	-17.570		257	Ala	N	H	-10.320
122	His	N	H	6.380		259	Phe	N	H	-7.420
125	Thr	N	H	-12.330		260	Lys	N	H	-15.000

Table S2: H-bond restraints for strong H-bonds identified through HDX, according to Table S4 and Fig. S20 and S21. The first column shows the amide proton while the second one shows the oxygen. (Acceptor residues were derived from the hCAII crystal structure 2CBA coordinates using HBOND, cib.cf.ocha.ac.jp/bitool/HBOND.) Amide protons 199 and 213 seem to have no binding partner in 2cba. Column 3 to 5 show the restraint value for structure calculation.

Residue	nucleus 2	Distance	error	error
57	69 CO	1.9 Å	+0.5 Å	-0.2 Å
58	69 CO	1.9 Å	+0.5 Å	-0.2 Å
64	62 OD1 Asn	1.9 Å	+0.5 Å	-0.2 Å
67	60 CO	1.9 Å	+0.5 Å	-0.2 Å
98	115 CO	1.9 Å	+0.5 Å	-0.2 Å
112	109 CO	1.9 Å	+0.5 Å	-0.2 Å
114	105 OG Serin	1.9 Å	+0.5 Å	-0.2 Å
125	87 CO	1.9 Å	+0.5 Å	-0.2 Å
127	124 CO	1.9 Å	+0.5 Å	-0.2 Å
133	129 CO	1.9 Å	+0.5 Å	-0.2 Å
134	132 CO	1.9 Å	+0.5 Å	-0.2 Å
136	133 CO	1.9 Å	+0.5 Å	-0.2 Å
139	138 CO	1.9 Å	+0.5 Å	-0.2 Å
160	156 CO	1.9 Å	+0.5 Å	-0.2 Å
184	181 CO	1.9 Å	+0.5 Å	-0.2 Å
223	219 CO	1.9 Å	+0.5 Å	-0.2 Å
248	30 CO	1.9 Å	+0.5 Å	-0.2 Å

Table S3: NMR structural statistics for the ensemble of 20 refined conformers of hCAII (*pdb id:6HD2*)

**Conformationally restricting restraints**[a]	
**Distance Restraint List**	
Total	4550
Intraresidue (i = j)	764
Sequential (| i-j | = 1)	1098
medium-range (2 ≤ | i-j | ≥ 4)	676
long-range (| i-j | ≥ 5)	1354
Hydrogen bonds	156
Dihedral angle restrains (ϕ and φ)	387
$^{1}H/^{15}N$-RDCs	115
No. of restraints per residue[b]	17.5
**Model quality**[c]	
Rmsd backbone atoms (Å)	0.89
Rmsd heavy atoms (Å)	2.2
Rmsd bond lengths (Å)	0.005
Rmsd bond angles (°)	0.8
**MolProbity Ramachandran statistics**[d]	
Most favored region (%)	83.6
Allowed region (%)	15.6
Additionally allowed region (%)	0.5
Disallowed region (%)	0.6
**Global quality scores (raw/Z score)**	
Verify3D	0.42
PROCHECK (ϕ-ψ)[e]	-0.61
PROCHECK (all)[e]	-0.46
MolProbity clash score	26.03
**Model contents**	
Total no. of residues	260
BMRB accession number	34308
PDB ID code	6HD2

[a] Analyzed for residues 1 to 260
[b] There are 254 residues with conformationally restricting constraints
[c] *Ordered residue ranges: 13-20,33-41,45-62,64-83,87-96,107-109,112-128,130-138,140-149,152-183,188-195,204-245,247-251,254-259*
[d] Residues selected based on: Dihedral angle order parameter, with S(ϕ)+S(ψ)>=1.8
[e] Residues with sum of phi and psi order parameters > 1.8
Generated using PSVS 1.5

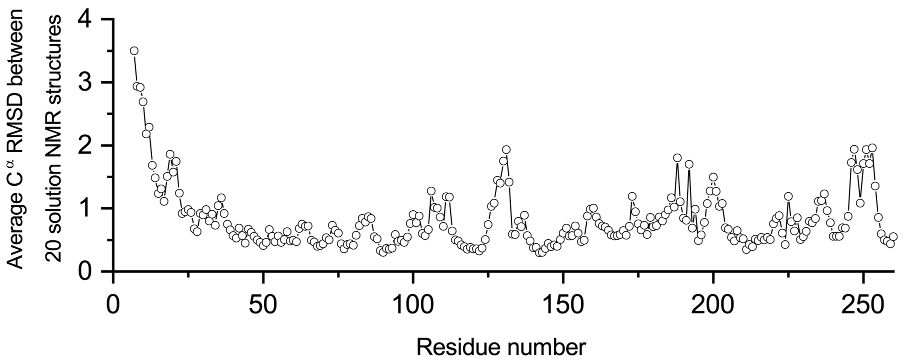

Fig. S7: Residue-resolved RMSD of C$^\alpha$ positions over a bundle of 20 lowest-energy structures.

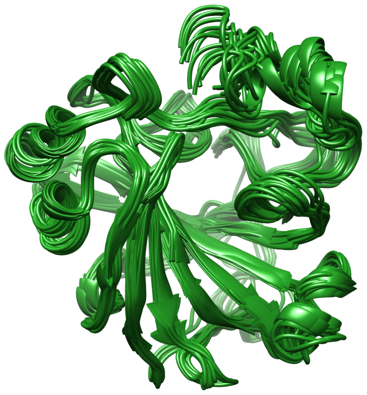

Fig. S8: hCAII monomeric structure in solution, shown as ensemble of 20 superimposed minimum-energy structures of monomeric hCAII. The N-terminal amino acids 1-7 are not shown.

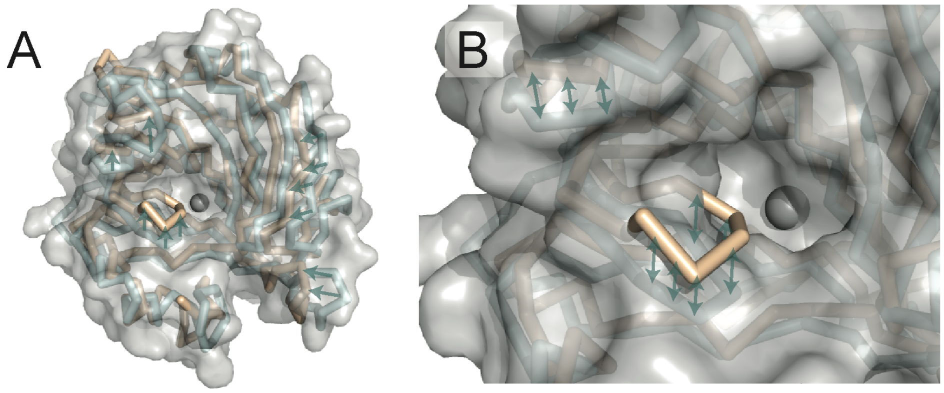

Fig. S9: Comparison of average-NMR-structure active-site-loop conformation with the conformation in the crystal (2cba). Green arrows denote changes going from X-ray to NMR structure. **A)** Overview, **B)** zoom into the active site.

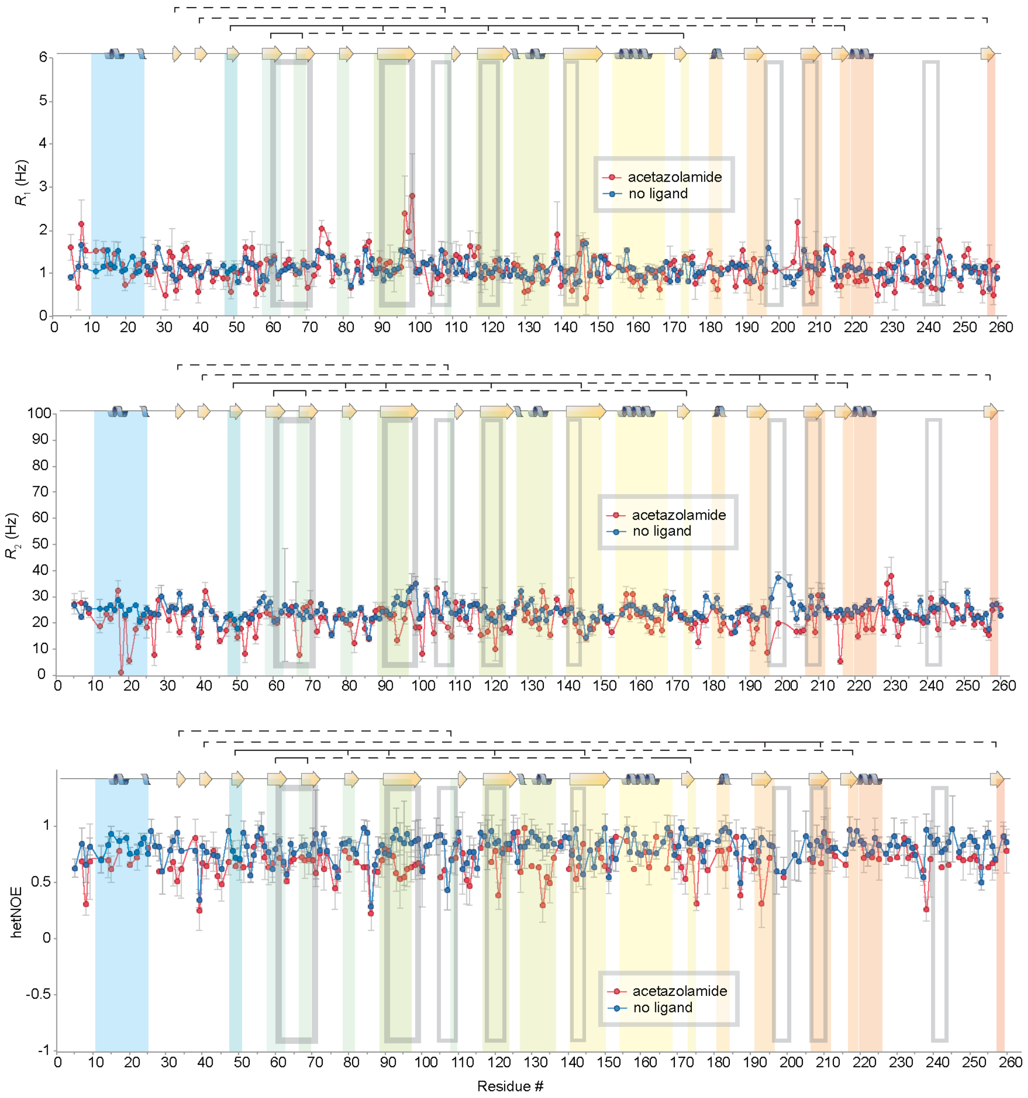

Fig. S10: Site-specific ^{15}N relaxation properties (R_1, R_2, and hetNOE) of non-ligand-bound (blue) and acetazolamide-bound hCAII (red).

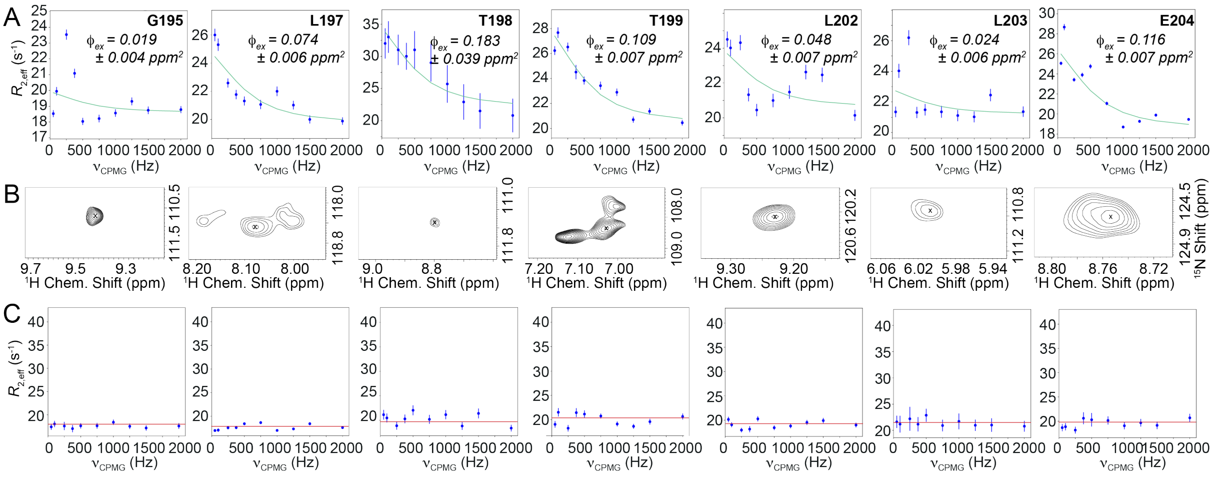

Fig. S11: Relaxation dispersion profiles **(A)** and peak shapes **(B)** for the active-site residues 195 to 204 at a temperature of 45 °C. (Residues 200 and 201 are prolines.) The exchange life time was globally fitted to τ_{ex} = 270 ± 13 µs. **C)** RD profiles for the inhibited case.

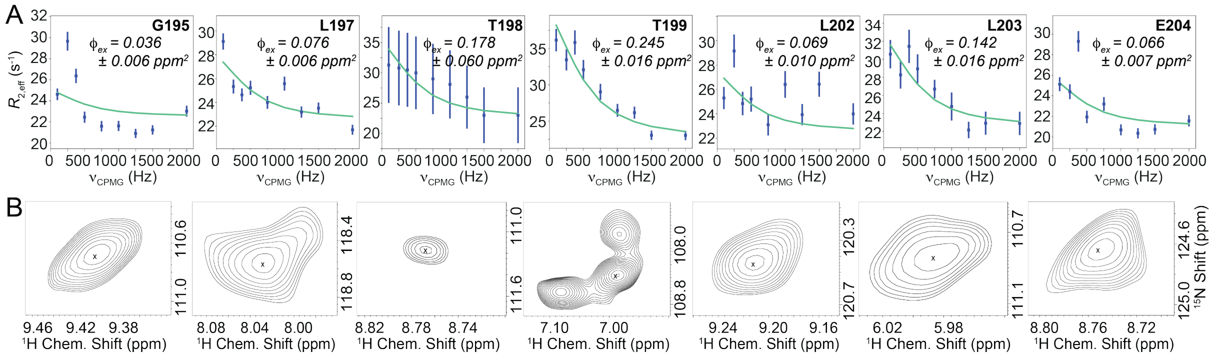

Fig. S12: Relaxation dispersion profiles **(A)** and peak shapes **(B)** for the active-site residues as in Fig. S10, but at 37 °C. The exchange life time was globally fitted to τ_{ex} = 290 ± 23 µs.

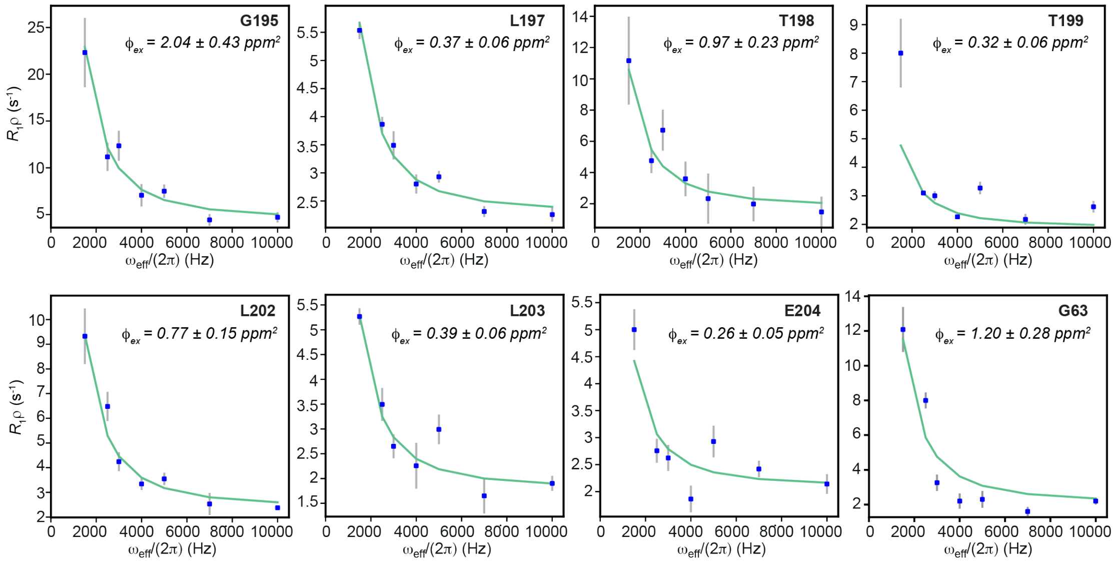

Fig. S13: Solid-state NMR relaxation dispersion of the active-site loop, with an exchange life time globally fit to 204 ± 46 μs. G63 was fit individually, yielding an exchange life time of 250 ± 67 μs.

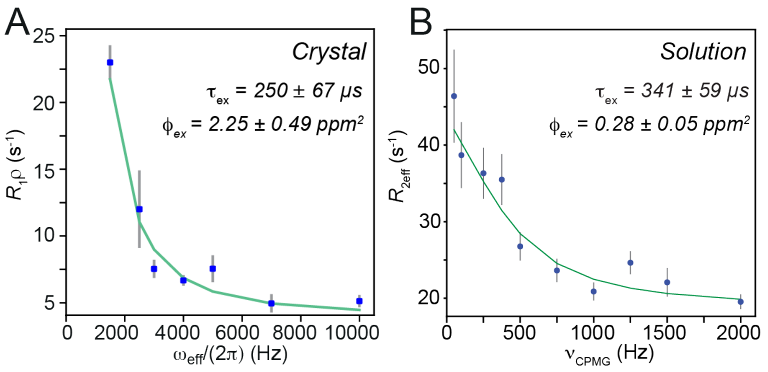

Fig. S14: Comparison of G6 dispersion profiles in crystalline form **(A)** and in solution at 45 °C **(B)**.

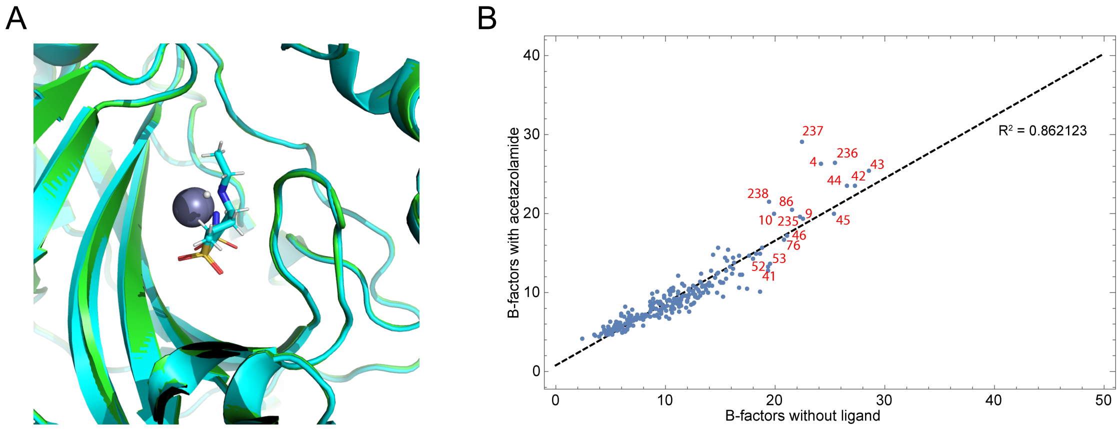

Fig. S15: A) Overlay of non-liganded (green, pdb 4Y0J) and dorzolamide-bound X-ray structures (blue, pdb 6BC9) of hCAII. **B)** Correlation between C$^{\alpha}$ B-factors of non-liganded (pdb 2cba) hCAII and hCAII soaked with acetazolamide (pdb 3hs4). Flexible residues with B-factors larger than 20 in any of the two structures are labeled. (None of them associated with the active-site loop.)

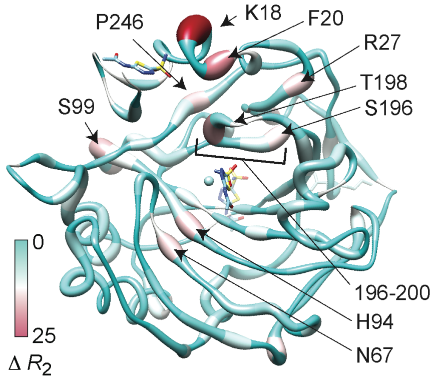

Fig. S16: Sausage representation of R_2 difference values between holo and AZ-bound hCAII.

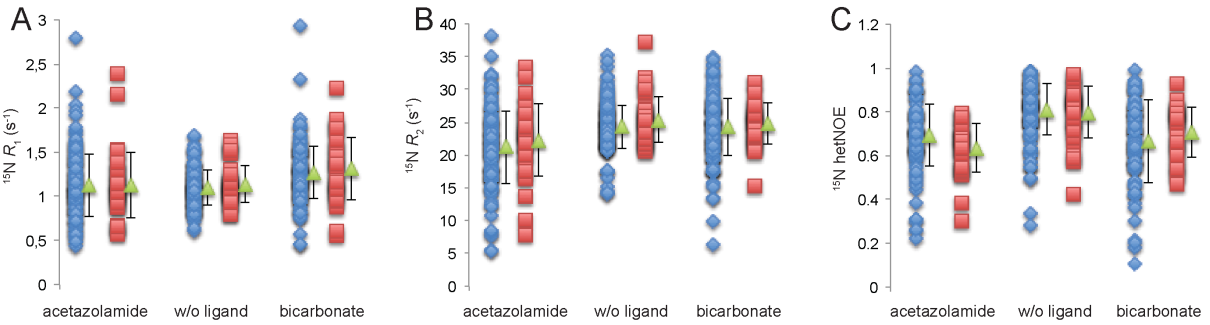

Fig. S17: Distributions of relaxation values compared between different ligand binding situations. **A)** ^{15}N R_1 relaxation, **B)** ^{15}N R_2 relaxation, **C)** ^{1}H-^{15}N het-nOe. Red and blue distributions denote active-site and non-active-site residues, respectively. Green triangles denote the average and standard deviation of each distribution.

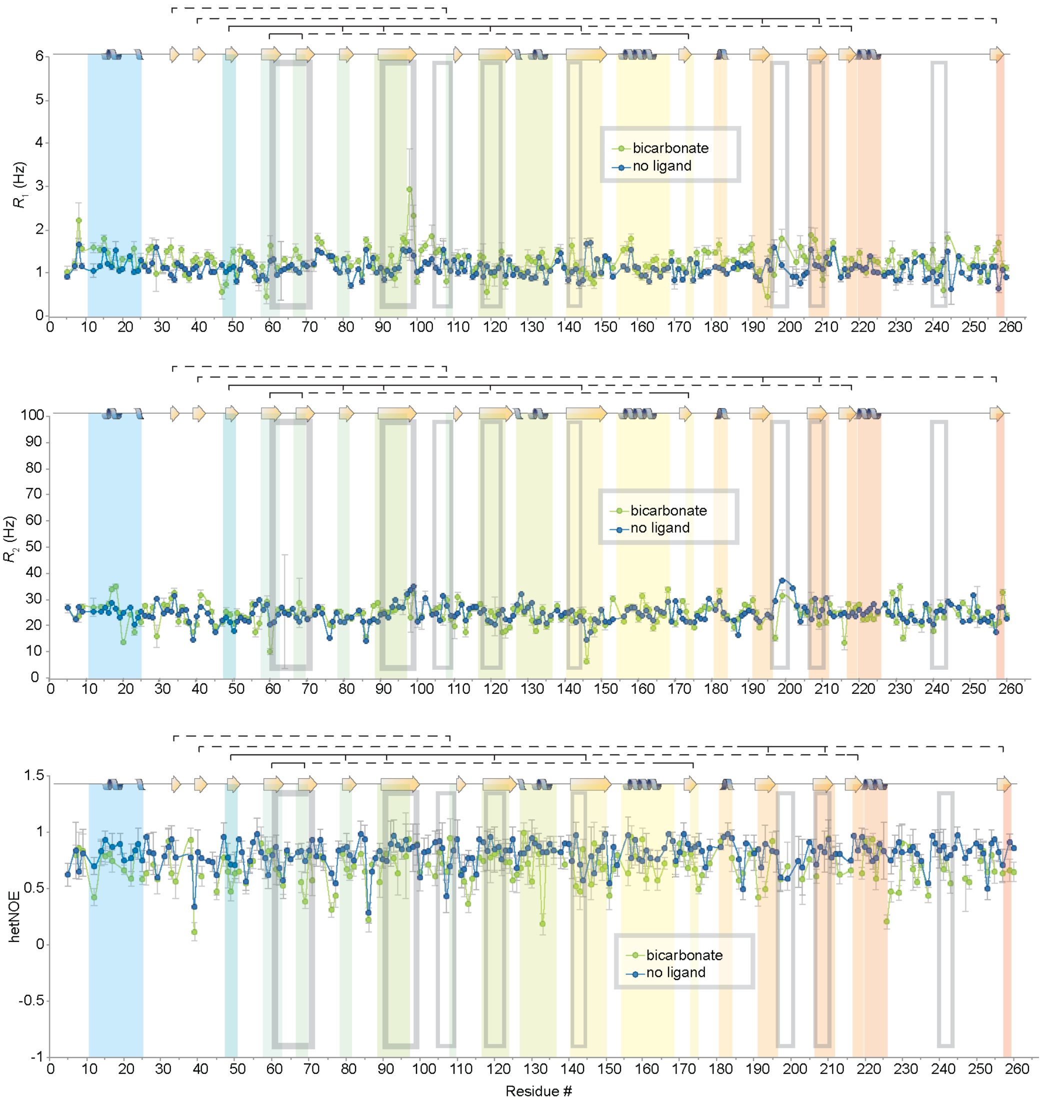

Fig. S18: Site-specific ^{15}N relaxation properties (R_1, R_2, and hetNOE) of non-ligand-bound (blue, as in Fig. S9) vs. bicarbonate-bound hCAII (green).

Assessment of protection of active-site regions of hCAII against H/D exchange

Site-resolved structural stability in (native) hCAII was assessed via a combination of H/D exchange experiments, in which lyophilized, protonated protein was dissolved in D_2O and – while protons steadily exchanged against deuterons – monitored as a function of time using HSQC experiments. The amide protons in hCAII can be divided into three main classes according to their exchange properties: *i)* The rapidly exchanging amide protons (red in Fig. S19) are, as expected, mainly located in loop regions and on the surface of the protein. More importantly, the exchange rates of the buried amides depend on both H-bond opening and accessibility of the site by breathing[18d, 26]. Here, we find *ii)* regions which show slow exchange (half-life times between a few hours to several days, turquoise in Fig. S19B), as well as *iii)*

residues that are fully protected and do not exchange over several months (W191 H^N in Fig S19A and blue color in Fig. S19B). Most group-ii residues show a double-exponential H/D exchange profile, representing the two components, H-bond opening and diffusion to the site. (Table S4 shows exchange rates obtained from mono- or biexponential fitting. Fig. S20 and S21 show the faster exponential rate as a function of sequence.) Interestingly, group-iii residues are localized close to both the active site as well as to the hydrogen-bonded water networks of hCAII [27]. (Fig. S22 depicts the presence of double exponentiality as a function of residue.) The intermediate lifetime of these amide protons – despite their direct proximity to the cone surface – confirms exchangeability within the pocket. T199 is shown in Fig. S19A in comparison to an exchange-protected residue further inside the core.

Also H64, which has been implicated in conformational dynamics, displays a similar amide exchange behavior. The sidechain of H64 at the entrance to the active site shows two orientations in various X-ray structures under cryogenic conditions. On the basis of ^{15}N relaxation dispersion (see main text), the residues around H64 backbone amide display dynamics on the 100 μs timescale both with and without inhibitor. Despite the exposed nature at the tip of a loop pointing to the bulk water, H/D exchange of the H64 amide is on the order of 1d (Fig. S19A). Accordingly, as expected, its H-bond is not broken by the exchange dynamics of the pocket architecture.

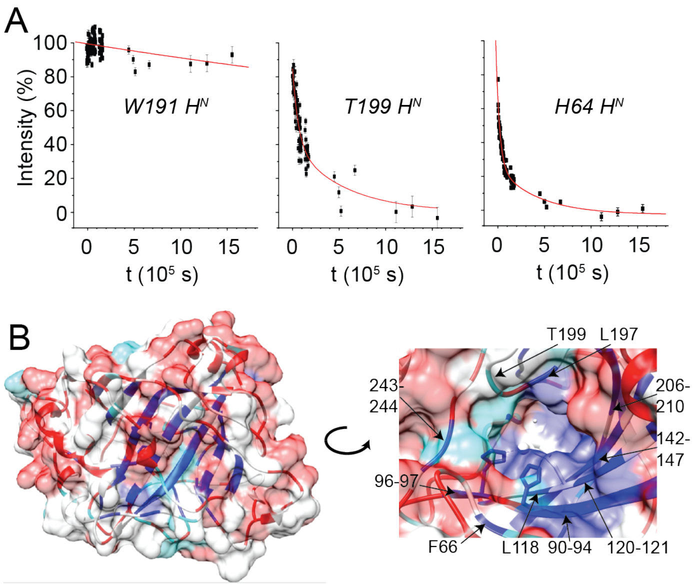

Fig. S19: hCAII active-site HD exchange in solution NMR. **A)** Slow H/D exchange profile of W191 H^N in comparison to intermediate rates in T199 H^N and H64 H^N. **B)** Water accessibility depicted on crystal structure 2CBA. Deep blue colored residues: no signal decay during the measured time period; turquoise residues: clearly double exponential behavior; red colored residues: either purely fast monoexponential behavior or equilibration before the first spectrum was measured. Right: close-up of the active site.

Table S4: Exponential rates fitted using eq. 1. The faster exponential rate is interpreted as the opening rate of the individual H-bond. In order to analyze the faster rate qualitatively we set a border of $2{\cdot}10^{-3}$ min^{-1}. Residues corresponding to rates slower than this are colored in blue, residues corresponding to rates faster than this are colored in yellow. Residues corresponding to peaks that have reached equilibrium before the first measurement (peaks gone "missing") are colored in red. Residues with very slow decay, such that a fit using eq. 1 is not possible, are shaded in gray.

Residue	H-bond opening rate k_1 [min^{-1}]	Second exponential rate k_2 [min^{-1}]
5	Missing	Missing
6	Missing	Missing
7	Missing	Missing
8	Missing	Missing
16*	5,26E-06	5,25E-06
17	Missing	Missing
20	Missing	Missing
25	Missing	Missing
28	0,00232	4,83E-05
29	2,19E-50	2,14E-50
31	1,40E-05	1,39E-05
33	7,52E-06	7,50E-06
34	Missing	Missing
37	Missing	Missing
38	Missing	Missing
39	Missing	Missing
41	Missing	Missing
43	Missing	Missing
44	Missing	Missing
45	Missing	Missing
49	Missing	Missing
50	Missing	Missing
53	Missing	Missing
54	Missing	Missing
56	(2,91E-05) missing peak, noise level	(2,91E-05) missing peak, noise level
57	0,00179	3,40E-04
58	1,70E-04	1,70E-04
59	1,70E-06	1,70E-06
61	0,01116	1,15E-137
62	Missing	Missing
63	Missing	Missing
64	0,00162	1,71E-04
66	6,82E-06	6,82E-06
67	0,0017	1,57E-04
69	8,32E-63	1,10E-64
72	Missing	Missing
74	Missing	Missing
76	Missing	Missing
81	Missing	Missing
82	Missing	Missing
84	Missing	Missing
86	Missing	Missing
87	Missing	Missing
88	0,00337	1,33E-22

Residue	H-bond opening rate k_1 [min^{-1}]	Second exponential rate k_2 [min^{-1}]
93	-7,76E-07	-7,95E-07
94	9,17E-50	1,37E-50
95	Missing	Missing
96	3,68E-39	3,13E-39
97	5,51E-44	5,51E-44
98	0,00104	1,27E-84
99	Missing	Missing
100	7,50E-05	7,50E-05
101	0,01549	1,02E-04
102	Missing	Missing
103	Missing	Missing
104	Missing	Missing
105	-8,23E-147	-8,23E-147
106 *	3,94E-06	3,94E-06
107	3,36E-50	1,06E-57
108	1,07E-06	1,06E-06
109	5,53E-05	5,53E-05
110	0,00667	0,00667
111	Missing	Missing
112	0,00138	1,67E-04
113	Missing	Missing
114	0,0015	2,10E-04
115	Missing	Missing
116	4,14E-06	4,13E-06
117 *	1,81E-6	1,81E-6
118	1,93E-06	1,93E-06
(119) *	(3,70E-33)	(9,50E-38)
120	-	2,55E-05
121	2,43E-06	2,41E-06
123	1,04E-05	1,04E-05
124	6,22E-06	6,22E-06
125	0,00206	2,11E-04
126	Missing	Missing
127	0,00133	1,78E-04
128	Missing	Missing
130	Missing	Missing
131	Missing	Missing
132	Missing	Missing
133	0,00152	4,50E-05
134	1,44E-04	-
135	0,03984	0,00343
136	0,00167	1,00E-04
138	Missing	Missing
139	0,00168	0,00168

90	4,43E-05	4,43E-05
91	7,05E-06	7,04E-06
92	5,93E-39	2,83E-39
144	2,01E-49	1,35E-49
145	5,80E-36	1,97E-36
146	3,44E-06	3,44E-06
147	8,91E-06	8,89E-06
150	Missing	Missing
153	1,83E-66	1,83E-66
156	Missing	Missing
157	Missing	Missing
159	0,03158	0,03158
160	1,02E-04	-
161	0,02679	-5,92E-117
163	Missing	Missing
164	1,99E-04 missing peak, noise level	1,99E-04 missing peak, noise level
165	Missing	Missing
166	0,00281	1,28E-86
167	0,00371	1,98E-04
168	0,00819 missing peak, noise level	0,00818 missing peak, noise level
169	Missing	Missing
171	Missing	Missing
172	Missing	Missing
175	Missing	Missing
176	Missing	Missing
179	Missing	Missing
181	(7,83E-05) missing peak, noise level	(3,30E-155) missing peak, noise level
182	Missing	Missing
183	0,00372	6,39E-05
184	1,90E-04	1,90E-04
187	Missing	Missing
188	Missing	Missing
191	5,86E-06	5,85E-06
192	1,79E-06	1,79E-06
193	7,81E-07	7,68E-07
195	-3,08E-154	-8,48E-142
196 *	5,11E-49	5,11E-49
197	6,93E-33	2,18E-33
198	Missing	Missing
199	9,42E-04	9,97E-05
203	1,15E-90	2,81E-99
204	Missing	Missing
205	0,00366	1,32E-04
206	2,95E-05	2,95E-05
207	4,07E-06	4,06E-06
208	3,36E-40	2,17E-40
209 *	1,12E-05	1,12E-05
210	2,92E-06	2,92E-06
144	2,01E-49	1,35E-49
145	5,80E-36	1,97E-36
146	3,44E-06	3,44E-06

140	6,19E-30	1,94E-30
142	-1,42E-06	-1,42E-06
143	1,35E-52	3,68E-55
156	Missing	Missing
157	Missing	Missing
159	0,03158	0,03158
160	1,02E-04	-
161	0,02679	-5,92E-117
163	Missing	Missing
164	1,99E-04 missing peak, noise level	1,99E-04 missing peak, noise level
165	Missing	Missing
166	0,00281	1,28E-86
167	0,00371	1,98E-04
168	0,00819 missing peak, noise level	0,00818 missing peak, noise level
169	Missing	Missing
171	Missing	Missing
172	Missing	Missing
175	Missing	Missing
176	Missing	Missing
179	Missing	Missing
181	(7,83E-05) missing peak, noise level	(3,30E-155) missing peak, noise level
182	Missing	Missing
183	0,00372	6,39E-05
184	1,90E-04	1,90E-04
187	Missing	Missing
188	Missing	Missing
191	5,86E-06	5,85E-06
192	1,79E-06	1,79E-06
193	7,81E-07	7,68E-07
195	-3,08E-154	-8,48E-142
196 *	5,11E-49	5,11E-49
197	6,93E-33	2,18E-33
198	Missing	Missing
199	9,42E-04	9,97E-05
203	1,15E-90	2,81E-99
204	Missing	Missing
205	0,00366	1,32E-04
206	2,95E-05	2,95E-05
207	4,07E-06	4,06E-06
208	3,36E-40	2,17E-40
209 *	1,12E-05	1,12E-05
210	2,92E-06	2,92E-06
211	3,76E-07	3,73E-07
213	0,00149	1,78E-04
215	1,95E-06	1,94E-06
217	3,57E-06	3,57E-06
222*	5,42E-05	5,42E-05
223	1,39E-04	1,39E-04
224	0,0136	9,65E-05
227	0,0044	2,20E-97
229	0,00892	4,03E-84

147	8,91E-06	8,89E-06
150	Missing	Missing
153	1,83E-66	1,83E-66
233	(9,89E-05) already decayed	(9,89E-05) already decayed
235	Missing	Missing
238	Missing	Missing
239	Missing	Missing
240	2,71E-06	2,71E-06
241	0,02177	-5,71E-108
243	2,20E-05	2,20E-05
244	8,59E-06	8,58E-06
245	Missing	Missing
247	Missing	Missing
248	8,80E-04	3,70E-05
250	Missing	Missing
255	Missing	Missing
258	0,00238	5,802E-96
259	Missing	Missing
260	Missing	Missing

230	(6,76E-06) missing peak, noise level	(5,85E-06) missing peak, noise level
231	Missing	Missing
232	Missing	Missing

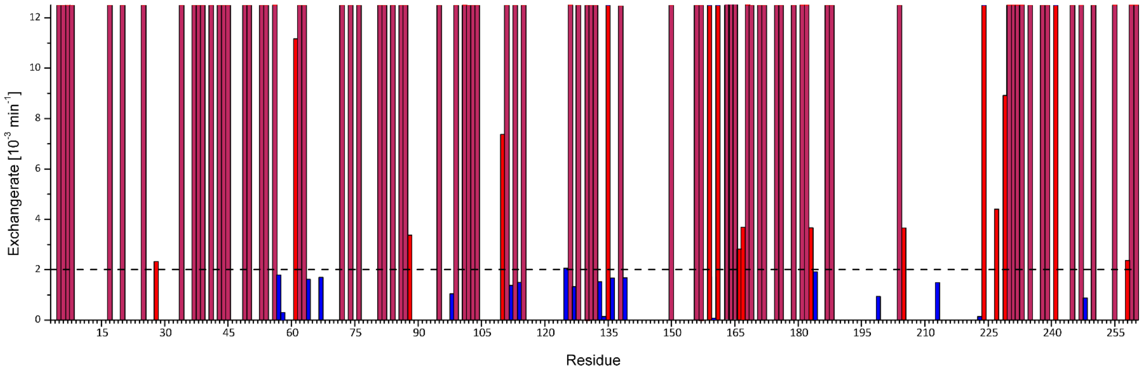

Fig. S20: Fast and intermediate H/D-exchanging residues. The faster exchange rate of the double-exponential function 1 is interpreted as the H-bond opening rate, corresponding to the H-bond strength. The dashed line marks an arbitrary border below which the residues are counted as H-bonded and are colored in blue. Residues with a rate faster than this are colored in red. Residues which are already fully decayed before the first measurement point are colored in ruby, they are not at all or weakly H-bonded. Very long-lived residues (gray in Table S4) cannot be fitted accurately and hence are not shown.

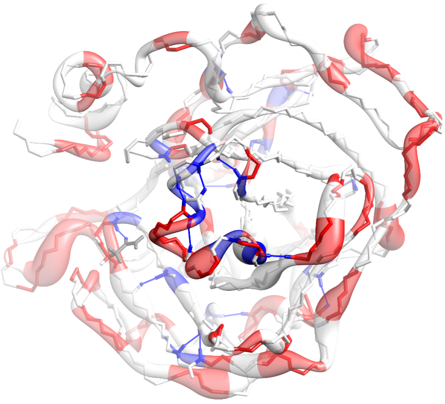

Fig. S21: H-bonds depicted on the crystal structure of hCAII. Strong H-bonds according to Table S4 and Fig. S20 are shown as blue lines. In this plot ^{15}N $R_{1\rho}$ relaxation data from crystalline hCAII[1] is shown as ribbon thickness. The ribbon of strongly H-bonded residues is colored in blue, while the ribbon of not or weakly H-bonded residues, according to the "missing" peaks in Table S4 and Fig. S20, is colored in red. Sidechains are colored in gray.

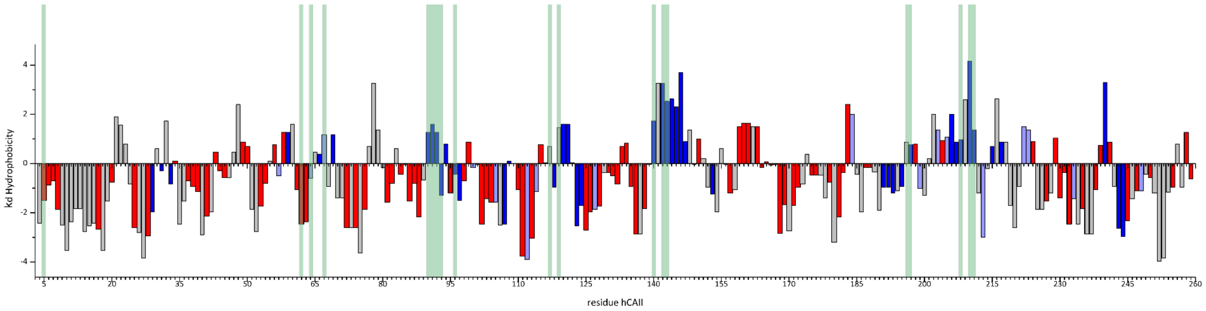

Fig. S22: Exchange-hindered residues (dark blue) and exchanging residues (red) as a function of sequence, presented with their Kite-Doolittle hydrophobicity as a moving average of 3. Exchange-hindered residues show nearly no signal decay during the measured time period. Light-blue-labeled residues show double exponential behavior according to Table S4. Red-labeled residues show either purely fast mono-exponential behavior or are already equilibrated before the first spectrum was obtained (dead time of around 15 min.). The green-shaded residues denote the active site.

References:

[1] S. K. Vasa, H. Singh, P. Rovó, R. Linser, *J. Phys. Chem. Lett.* **2018**, *9*, 1307−1311.

[2] E. Luchinat, L. Barbieri, M. Cremonini, A. Nocentini, C. T. Supuran, L. Banci, *Angew. Chem., Int. Ed.* **2020**, *59*, 6535-6539.

[3] H. Singh, S. K. Vasa, H. Jangra, P. Rovó, C. Päslack, C. K. Das, H. Zipse, L. V. Schäfer, R. Linser, *J. Am. Chem. Soc.* **2019**, *141*, 19276-19288.

[4] S. K. Vasa, H. Singh, K. Grohe, R. Linser, *Angew. Chem., Int. Ed.* **2019**, *58*, 5758-5762.

[5] R. A. Venters, B. T. Farmer, C. A. Fierke, D. S. Leonard *J. Mol. Biol.* **1996**, *264*, 1101–1116.

[6] M. Ottiger, F. Delaglio, A. Bax, *J. Magn. Reson.* **1998**, *131*, 373-378.

[7] M. Zweckstetter, *Nat. Protoc.* **2008**, *3*, 679-690.

[8] S. Meiboom, D. Gill, *Rev. Sci. Inst.* **1958**, *29*, 688-691.

[9] R. Keller, PhD thesis (ETH Zurich), **2004**.

[10] W. F. Vranken, W. Boucher, T. J. Stevens, R. H. Fogh, A. Pajon, P. Llinas, E. L. Ulrich, J. L. Markley, J. Ionides, E. D. Laue, *Proteins* **2005**, *59*, 687–696.

[11] F. A. A. Mulder, N. R. Skrynnikov, B. Hon, F. W. Dahlquist, L. E. Kay, *J. Am. Chem. Soc.* **2001**, *123*, 967-975.

[12] W. Lee, M. Tonelli, J. L. Markley, *Bioinformatics* **2015**, *31*, 1325-1327.

[13] M. Bieri, P. R. Gooley, *BMC Bioinformatics* **2011**, *12*, 421.

[14] A. Mazur, B. Hammesfahr, C. Griesinger, D. Lee, M. Kollmar, *Bioinformatics* **2013**, *29*, 1819-1820.

[15] Y. Shen, F. Delaglio, G. Cornilescu, A. Bax, *J. Biomol. NMR* **2009**, *44*, 213-223.

[16] W. Rieping, M. Habeck, B. Bardiaux, M. Bernard, T. E. Malliavin, M. Nilges, *Bioinformatics* **2007**, *23*, 381-382.

[17] A. T. Brünger, *Nat. Protoc.* **2007**, *2*, 2728-2733.

[18] a) S. W. Englander, D. B. Calhoun, J. J. Englander, N. R. Kallenbach, R. K. Liem, E. L. Malin, C. Mandal, J. R. Rogero, *Biophys. J.* **1980**, *32*, 577-589; b) O. Jardetzky, M. D. Finucane, *Molecular Physics* **1998**, *95*, 1127-1136; c) A. Tomita, T. Sato, K. Ichiyanagi, S. Nozawa, H. Ichikawa, M. Chollet, F. Kawai, S.-Y. Park, T. Tsuduki, T. Yamato, S.-y. Koshihara, S.-i. Adachi, *Proc. Natl. Acad. Sci. U.S.A.* **2009**, *106*, 2612-2616; d) K. Grohe, K. T. Movellan, S. K. Vasa, K. Giller, S. Becker, R. Linser, *J. Biomol. NMR* **2017**, *68*, 7-17.

[19] M. J. Abraham, T. Murtola, R. Schulz, S. Páll, J. C. Smith, B. Hess, E. Lindahl, *SoftwareX* **2015**, *1-2*, 19-25.

[20] B. S. Avvaru, C. U. Kim, K. H. Sippel, S. M. Gruner, M. Agbandje-McKenna, D. N. Silverman, R. McKenna, *Biochemistry* **2009**, *49*, 249-251.

[21] M. A. Pinard, C. D. Boone, B. D. Rife, C. T. Supuran, R. McKenna, *Bioorg. Med. Chem.* **2013**, *21*, 7210-7215.

[22] K. T. Debiec, D. S. Cerutti, L. R. Baker, A. M. Gronenborn, D. A. Case, L. T. Chong, *J. Chem. Theory Comput.* **2016**, *12*, 3926-3947.

[23] a) P. Li, K. M. Merz, *J. Chem. Inf. Model.* **2016**, *56*, 599-604; b) M. B. Peters, Y. Yang, B. Wang, L. Füsti-Molnár, M. N. Weaver, K. M. Merz, *J. Chem. Theory Comput.* **2010**, *6*, 2935-2947.

[24] U. Essmann, L. Perera, M. L. Berkowitz, T. Darden, H. Lee, L. G. Pedersen, *J. Chem. Phys.* **1995**, *103*, 8577–8592.

[25] G. Bussi, D. Donadio, M. Parrinello, *J. Chem. Phys. Lett.* **2007**, *126*, 014101.

[26] C. J. Craven, N. M. Derix, J. Hendriks, R. Boelens, K. J. Hellingwerf, R. Kaptein, *Biochemistry* **2000**, *39*, 14392-14399.

[27] K. M. Merz Jr., M. A. Murcko, P. A. Kollman, *J. Am. Chem. Soc.* **1991**, *113*, 406-411.
